# Chronic Lung Disease as a Risk Factor for Long COVID in Patients Diagnosed With Coronavirus Disease 2019: A Retrospective Cohort Study

**DOI:** 10.1093/ofid/ofae424

**Published:** 2024-08-23

**Authors:** Xiaotong Zhang, Alfred Jerrod Anzalone, Daisy Dai, Gary Cochran, Ran Dai, Mark E Rupp, for the National COVID Cohort (N3C) Collaborative, Adam B Wilcox, Adam M Lee, Alexis Graves, Alfred (Jerrod) Anzalone, Amin Manna, Amit Saha, Amy Olex, Andrea Zhou, Andrew E Williams, Andrew Southerland, Andrew T Girvin, Anita Walden, Anjali A Sharathkumar, Benjamin Amor, Benjamin Bates, Brian Hendricks, Brijesh Patel, Caleb Alexander, Carolyn Bramante, Cavin Ward-Caviness, Charisse Madlock-Brown, Christine Suver, Christopher Chute, Christopher Dillon, Chunlei Wu, Clare Schmitt, Cliff Takemoto, Dan Housman, Davera Gabriel, David A Eichmann, Diego Mazzotti, Don Brown, Eilis Boudreau, Elaine Hill, Elizabeth Zampino, Emily Carlson Marti, Emily R Pfaff, Evan French, Farrukh M Koraishy, Federico Mariona, Fred Prior, George Sokos, Greg Martin, Harold Lehmann, Heidi Spratt, Hemalkumar Mehta, Hongfang Liu, Hythem Sidky, J W Awori Hayanga, Jami Pincavitch, Jaylyn Clark, Jeremy Richard Harper, Jessica Islam, Jin Ge, Joel Gagnier, Joel H Saltz, Joel Saltz, Johanna Loomba, John Buse, Jomol Mathew, Joni L Rutter, Julie A McMurry, Justin Guinney, Justin Starren, Karen Crowley, Katie Rebecca Bradwell, Kellie M Walters, Ken Wilkins, Kenneth R Gersing, Kenrick Dwain Cato, Kimberly Murray, Kristin Kostka, Lavance Northington, Lee Allan Pyles, Leonie Misquitta, Lesley Cottrell, Lili Portilla, Mariam Deacy, Mark M Bissell, Marshall Clark, Mary Emmett, Mary Morrison Saltz, Matvey B Palchuk, Melissa A Haendel, Meredith Adams, Meredith Temple-O’Connor, Michael G Kurilla, Michele Morris, Nabeel Qureshi, Nasia Safdar, Nicole Garbarini, Noha Sharafeldin, Ofer Sadan, Patricia A Francis, Penny Wung Burgoon, Peter Robinson, Philip R O Payne, Rafael Fuentes, Randeep Jawa, Rebecca Erwin-Cohen, Rena Patel, Richard A Moffitt, Richard L Zhu, Rishi Kamaleswaran, Robert Hurley, Robert T Miller, Saiju Pyarajan, Sam G Michael, Samuel Bozzette, Sandeep Mallipattu, Satyanarayana Vedula, Scott Chapman, Shawn T O’Neil, Soko Setoguchi, Stephanie S Hong, Steve Johnson, Tellen D Bennett, Tiffany Callahan, Umit Topaloglu, Usman Sheikh, Valery Gordon, Vignesh Subbian, Warren A Kibbe, Wenndy Hernandez, Will Beasley, Will Cooper, William Hillegass, Xiaohan Tanner Zhang

**Affiliations:** Department of Pharmacy, Xuanwu Hospital, Capital Medical University, Beijing, China; Department of Biostatistics, College of Public Health, University of Nebraska Medical Center, Omaha, Nebraska, USA; College of Pharmacy, University of Nebraska Medical Center, Omaha, Nebraska, USA; Department of Neurological Sciences, University of Nebraska Medical Center, Omaha, Nebraska, USA; Department of Biostatistics, College of Public Health, University of Nebraska Medical Center, Omaha, Nebraska, USA; College of Pharmacy, University of Nebraska Medical Center, Omaha, Nebraska, USA; Department of Biostatistics, College of Public Health, University of Nebraska Medical Center, Omaha, Nebraska, USA; Division of Infectious Diseases, Department of Internal Medicine, University of Nebraska Medical Center, Omaha, Nebraska, USA

**Keywords:** chronic lung disease, long COVID, SARS-CoV-2 infection, postacute sequelae of COVID, long COVID risk factor

## Abstract

**Background:**

Patients with coronavirus disease 2019 (COVID-19) often experience persistent symptoms, known as postacute sequelae of COVID-19 or long COVID, after severe acute respiratory syndrome coronavirus 2 (SARS-CoV-2) infection. Chronic lung disease (CLD) has been identified in small-scale studies as a potential risk factor for long COVID.

**Methods:**

This large-scale retrospective cohort study using the National COVID Cohort Collaborative data evaluated the link between CLD and long COVID over 6 months after acute SARS-CoV-2 infection. We included adults (aged ≥18 years) who tested positive for SARS-CoV-2 during any of 3 SARS-CoV-2 variant periods and used logistic regression to determine the association, considering a comprehensive list of potential confounding factors, including demographics, comorbidities, socioeconomic conditions, geographical influences, and medication.

**Results:**

Of 1 206 021 patients, 1.2% were diagnosed with long COVID. A significant association was found between preexisting CLD and long COVID (adjusted odds ratio [aOR], 1.36). Preexisting obesity and depression were also associated with increased long COVID risk (aOR, 1.32 for obesity and 1.29 for depression) as well as demographic factors including female sex (aOR, 1.09) and older age (aOR, 1.79 for age group 40–65 [vs 18–39] years and 1.56 for >65 [vs 18–39] years).

**Conclusions:**

CLD is associated with higher odds of developing long COVID within 6 months after acute SARS-CoV-2 infection. These data have implications for identifying high-risk patients and developing interventions for long COVID in patients with CLD.

## Teaser

This large-scale retrospective cohort study reveals a significant association between chronic lung disease and the postacute sequelae of COVID-19, along with obesity, depression, female sex, and older age. The findings underscore the need for targeted interventions in these high-risk groups.

Coronavirus disease 2019 (COVID-19), caused by viral infection with severe acute respiratory syndrome coronavirus 2 (SARS-CoV-2), has affected millions of patients worldwide. In the United States (US), there have been >100 million confirmed cases of COVID-19, with >1 million deaths reported between January 2020 and March 2023 [1]. Some survivors present with persistent neurological, respiratory, or cardiovascular symptoms after the acute phase of the infection, regardless of the initial disease severity [2]. The Centers for Disease Control and Prevention (CDC) describes these long-term effects of SARS-CoV-2 infection as post-COVID conditions (PCCs), postacute sequelae of COVID-19 (PASC), or long COVID [3]. Per the CDC, long COVID consists of a wide range of new, returning, or ongoing health problems occurring ≥4 weeks after first being infected with SARS-CoV-2 [3]. A meta-analysis showed that long COVID is associated with poor quality of life [4]. A cross-sectional study conducted in the US in 2021–2022 found that 13.9% of US adults with a prior positive COVID-19 test reported continued symptoms >2 months after acute illness, representing 1.7% of US adults [5]. Based on data collected by the US Census Bureau and CDC in June 2022, 19% of adults who have had COVID-19 have persistent symptoms of long COVID, and 7.5% of adults in the US have symptoms that have lasted >3 months after first infection [6]. An estimated 2 million people in the United Kingdom self-reported long COVID symptoms as of 5 March 2023 [7]. Data from the Survey of Health, Ageing and Retirement in Europe's corona surveys (2020–2021) indicated a prevalence of 71.6% for long COVID with an average of 3 symptoms among the respondents aged ≥50 years in 27 countries across Europe [8]. Given the substantial prevalence of long COVID, subsequent compromised quality of life, and the stress exerted on the healthcare system, it is warranted to identify the risk factors for long COVID and vulnerable populations.

Small-scale studies have been conducted to identify risk factors for long COVID. Age above 40–50 years, female sex, belonging to an ethnic minority group, smoking, obesity, higher number of preexisting medical conditions, and socioeconomic deprivation have shown an association with greater risk of long COVID, while completion of primary vaccination series prior to infection shows protective effects [5, 6, 9]. Previous studies based on regional COVID-19 patients suggest that preexisting lung disease, such as chronic obstructive pulmonary disease (COPD) and asthma, are risk factors for long COVID [7, 9–13]. A cross-sectional study that focused on adults hospitalized with a diagnosis of acute COVID-19 during the first 2 waves in the United Kingdom found that preexisting lung disease was associated with a greater number of long COVID symptoms [7]. A cohort study in Italy demonstrated a significant association between obstructive lung disease and long COVID among 739 nonhospitalized healthcare workers confirmed COVID-19 positive [13]. These studies were based on select study populations and conducted within different time frames. Many of them relied on a limited sample size and focused on a narrower timeframe of SARS-CoV-2 infection. The lack of a shared and specific definition of long COVID, and limited confounding factors included in the analyses, also suggests the need for further evaluation of the association between preexisting chronic lung diseases (CLDs) and the occurrence of long COVID. More recently, a retrospective case-control study based on the National COVID Cohort Collaborative (N3C) established a machine learning–based PASC diagnosis with CLD as one of the predictors in the model [14]. Large, randomized controlled trials, such as the Longitudinal Study of COVID-19 Sequelae and Immunity (RECON-19) [15] and Researching COVID to Enhance Recovery (RECOVER) [16] studies, have focused on cohort characterization and symptom clustering. However, systematic and large-scale studies evaluating associations between CLD and long COVID after acute SARS-CoV-2 infection are still limited.

This study aimed to investigate the impact of preexisting CLD on the clinical diagnosis of long COVID and assess the risk factors for developing long COVID among adult patients with CLD using real-world data from a large, nationally sampled cohort. Based on the reviewed literature, we hypothesized that adult patients with preexisting CLD will exhibit a higher risk of long COVID compared to those without.

## METHODS

### Study Design

The analytical data were sourced from the N3C Data Enclave, which harmonizes electronic health record data from 75 institutions into a single common data model. The criteria for confirmed SARS-CoV-2 infection (or confirmed COVID-19 positive) were based on the *International Classification of Diseases, Tenth Revision* (*ICD-10*) diagnostic code for COVID-19 diagnosis (U07.1) or positive SARS-CoV-2 laboratory result (qualitative reverse-transcription polymerase chain reaction or antigen test). The follow-up period for the cohort was 6 months after the acute phase of infection, which was defined as 4 weeks following the COVID-19 confirmation date. Patients were included if they (1) were ≥18 years of age when confirmed COVID-19 positive and (2) were confirmed COVID-19 positive during any of 3 consecutive SARS-CoV-2 variant dominance periods, defined as pre–Delta wave (29 December 2020–14 June 2021), Delta wave (15 June 2021–29 November 2021), and Omicron wave (30 November 2021–15 June 2022) [17]. We excluded patients who (1) were lost to follow-up without a valid long COVID diagnosis reported; (2) who died directly from COVID-19 during acute phase (within the 30-day time window after the acute COVID-19 diagnosis); and (3) who had missing information.

### Outcome

Our outcome was the incidence of long COVID diagnosis during the 6-month follow-up period after the acute phase of SARS-CoV-2 infection. The diagnosis of long COVID was denoted by the *ICD-10* diagnostic code for long COVID (U09.9). Long COVID diagnoses recorded during the acute phase were considered invalid based on the definition of long COVID by the CDC [3].

### Risk Factors

The primary exposure of this study was CLD before COVID-19 diagnosis using a clinician- and informatician-validated computable definition [18]. It refers to chronic disorders that affect the lungs and other parts of the respiratory system, including the conditions of COPD, sleep-disordered breathing, and interstitial lung disease [19]. The most common symptoms are shortness of breath, coughing, wheezing, and tightness in the chest [19]. For this study, we identified a cohort with preexisting CLD before the first acute COVID-19 diagnosis in the N3C Data Enclave (the CLD group). In addition to the primary exposure, we included variables potentially associated with the outcome. Demographic characteristics were captured using age, sex, and race/ethnicity. Social history factors included tobacco use and substance abuse. Vaccination status was categorized into 3 groups: (1) incomplete vaccination (including patients who were unvaccinated or failed to complete the primary vaccination series, the reference); (2) completed primary series (including patients who completed the primary vaccination series but not boosted); and (3) boosted (including patients who received 1 or more boosters). We also accounted for the effects of medication history before the first SARS-CoV-2 infection and medications for acute COVID-19 treatment (see details in Supplementary Table 1), and all of the variables on medication use were dichotomous. The severity of infection was included as a 4-level ordinal variable in the analysis as well, ranging from “mild with no emergency department visit or hospitalization” to “use of intermittent mandatory ventilation or extracorporeal membrane oxygenation during COVID hospitalization.” Immune status after recovery was included using SARS-CoV-2 antibody status after infection; repeated SARS-CoV-2 infection following the initial infection was also considered as a potential risk factor for long COVID. Medical conditions were assessed with comorbidities including cardiovascular diseases, infectious diseases, neurological and cognitive disorders, and malignant tumors. We also adjusted for the effects of variant dominance periods and healthcare access based on geographic areas. All of the covariates mentioned above are categorical variables and were selected based on a priori knowledge of risk factors for long COVID [5, 9–11, 20–22]. In short, the confounders we adjusted for in our analysis include age, sex, race/ethnicity, tobacco usage, substance use disorder, COVID-19 vaccination status, medications, the severity of infection, SARS-CoV-2 antibody status after infection, reinfection, comorbidities, variant waves, and urban/rural residency. The summary statistics of all adjusted variables are provided in Supplementary Table 1.

### Statistical Analysis

We used descriptive statistics to show the distribution of demographic features, social history, health status, and medical conditions among the cohort patients and compared the distributions of these covariates between the cohort patients and those lost to follow-up (Supplementary Tables 1 and 2). We also used marginalized and conditional probabilities to demonstrate the incidence of long COVID and its distribution among stratified patient populations (Supplementary Table 3).

#### Primary Analyses

We assessed the association between CLD and long COVID with a multiple logistic regression model that adjusted for all the covariates, including demographic variables, social factors, immune status, medication use, and comorbidities. We computed adjusted odds ratios (aORs) with accompanying 95% confidence intervals (CIs) to evaluate the effects of underlying CLD on the incidence of long COVID. We also report aORs and 95% CIs for other risk factors.

#### Subgroup Analysis

We assessed the risk factors of long COVID among the patients with preexisting CLD, using multiple logistic regression, and report the aOR and 95% CI.

#### Sensitivity Analysis

Some of the patients with long COVID diagnoses in our cohort had a follow-up period of <6 months. Since we were unable to discern the reasons for the loss of follow-up from N3C and this heterogeneity might potentially cause bias, we removed these patients from the cohort and repeated the proposed primary analysis to check how it would influence our results. In addition, long COVID is potentially underreported in the N3C data. We randomly removed the diagnosis of a proportion of the confirmed long COVID cases from the cohort to simulate an underdiagnosis scenario and repeated the primary analysis to understand the robustness against the underreporting issue of our analysis. Death is a competing risk of long COVID, which potentially led to bias in the observation time for long COVID. We performed a sensitivity analysis by repeating the primary analysis on the cohort after removing all patients with post–acute COVID-19 death.

## RESULTS

### Study Population

**Figure 1.**
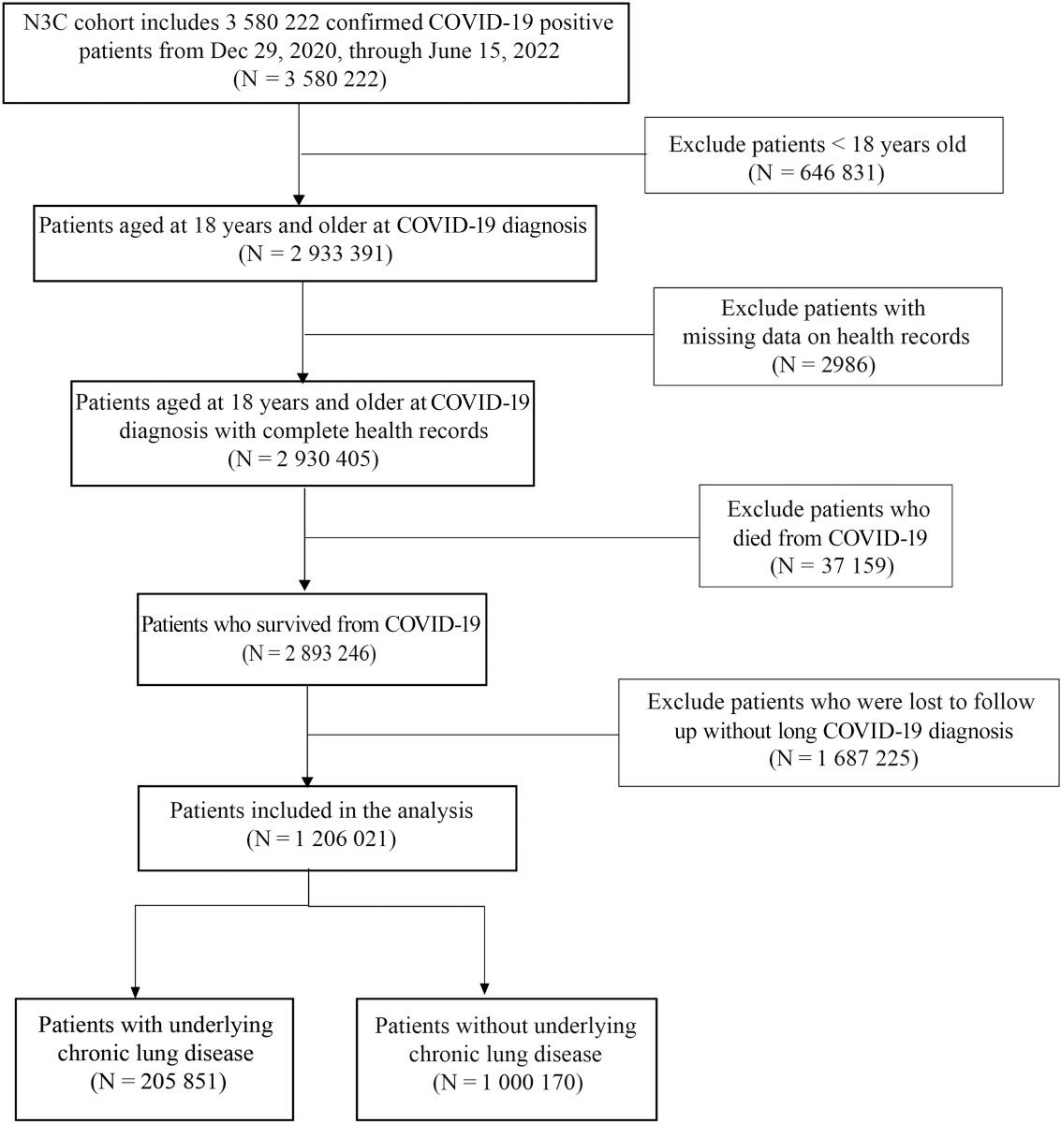
Flowchart detailing the inclusion and exclusion criteria applied in the construction of the analytical cohort from the National COVID Cohort Collaborative (N3C) observational electronic health record data. Abbreviation: COVID-19, coronavirus disease 2019.

We extracted data from N3C on 4 March 2023. A total of 3 580 222 patients who were confirmed COVID-19 positive during the time period from 29 December 2020 to 15 June 2022 were reported to N3C. Of these, 2 930 405 patients were adults (≥18 years of age) at COVID-19 diagnosis. A total of 2 893 246 patients survived the acute phase of infection. Among these survivors, 1 687 225 patients were lost to follow-up within the first 6 months without a long COVID diagnosis. We finally included 1 206 021 patients in the analysis. We divided the patient cohort into 2 groups (CLD group vs non-CLD group) based on the presence of underlying CLD (Figure 1).

### Descriptive Data

**Figure 2.**
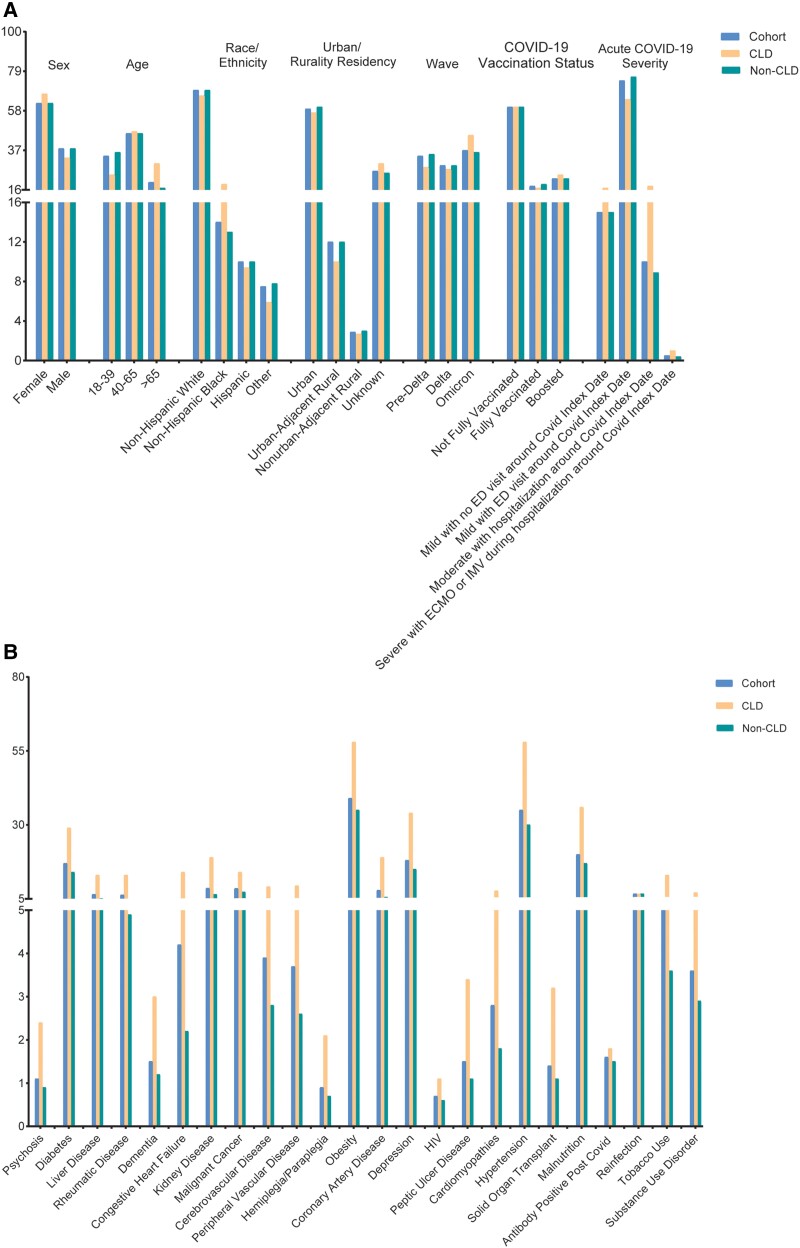
Bar plot depicting baseline characteristics distributions for the entire cohort (blue/left), individuals with chronic lung disease (CLD; orange/middle), and non-CLD participants (green/right). Characteristics include demographic data, socioeconomic factors, and geographical details (*A*) and existing comorbidities (*B*). Abbreviations: CLD, chronic lung disease; COVID-19, coronavirus disease 2019; ECMO, extracorporeal membrane oxygenation; ED, emergency department; HIV, human immunodeficiency virus; IMV, intermittent mandatory ventilation.

Nearly half (46%) of the patients in the cohort were aged 40–65 years. Almost two-thirds (62%) of the patients were female. Comparing the subcohort with preexisting CLD (CLD group) and the subcohort without preexisting CLD (non-CLD group), the CLD group had a higher percentage of elderly (>65 years old), female, and non-Hispanic Black patients. The number of confirmed COVID-19 cases across 3 consecutive SARS-CoV-2 variant dominance periods was comparable (pre-Delta vs Delta vs Omicron: 34% vs 29% vs 37%). Among the cohort, 17.1% of the patients had preexisting CLD. Baseline patient characteristics distributions are shown in Figure 2. Complete baseline patient characteristics are summarized in Supplementary Table 1. The patients lost to follow-up had similar baseline patient characteristics distributions as our study cohort, indicating the loss to follow-up mechanism being random. Therefore, there is very minor concern about biased results due to the exclusion of this cohort. Details are shown in Supplementary Table 2.

In the cohort, 1.2% of the patients were diagnosed with long COVID according to the *ICD-10* diagnostic code for long COVID (U09.9) during the follow-up period. Patients in the CLD group had a higher incidence of being diagnosed with long COVID compared to those in the non-CLD group (2.1% vs 1.1%). Each type of CLD, including COPD, asthma, and bronchiectasis, was related to higher incidence of long COVID (COPD: 2.1% vs 1.2%; asthma: 2.1% vs 1.1%; bronchiectasis: 3.2% vs 1.2%). The incidence of long COVID in the older age group was higher compared to the younger age group (>65 years vs 40–65 years vs 18–39 years: 1.5% vs 1.5% vs 0.7%). COVID-19 cases during Delta and Omicron wave showed higher incidences for long COVID compared to pre–Delta wave (pre-Delta vs Delta vs Omicron: 0.3% vs 1.7% vs 1.7%). Details of long COVID incidences are shown in Supplementary Table 3.

### Primary Analysis

**Figure 3.**
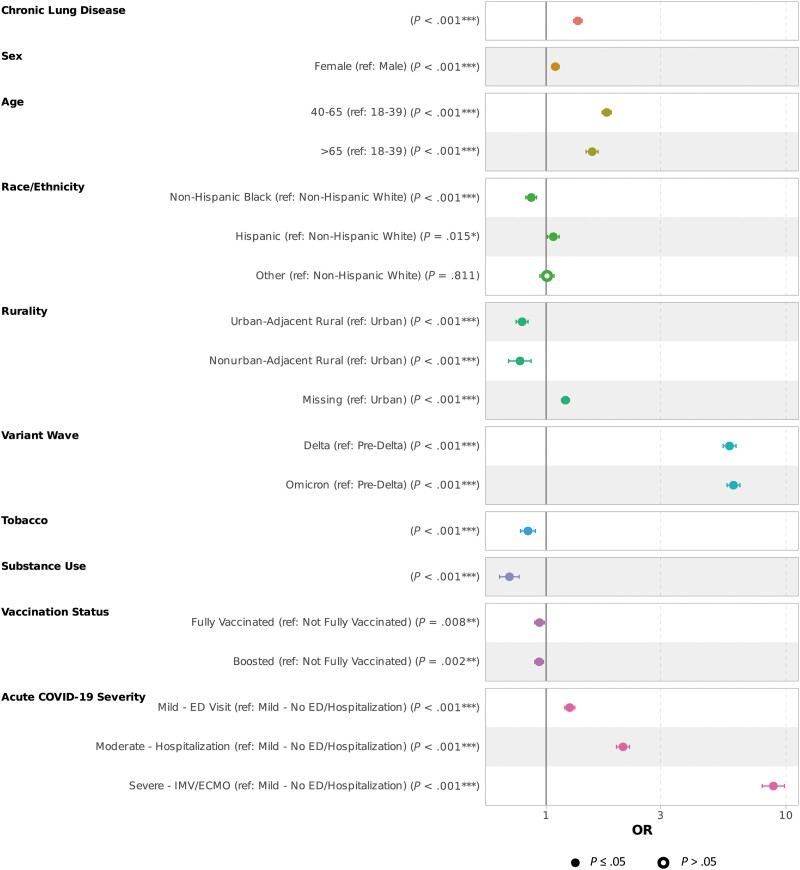
Forest plot depicting risk factors associated with long COVID with multiple logistic regression. Risk factors presented include demographic data, socioeconomic factors, and geographical details. *P < .05; **P < .005; ***P < .001. Abbreviations; COVID-19, coronavirus disease 2019; ECMO, extracorporeal membrane oxygenation; ED, emergency department; IMV, intermittent mandatory ventilation; OR, odds ratio.

From the multiple logistic regression adjusted for demographic, socioeconomic, and medical factors, CLD was significantly associated with long COVID. After adjustments, patients with CLD had 36% higher odds of long COVID than those without (adjusted odds ratio [aOR], 1.36 [95% CI, 1.3–1.41]). Among all the other covariates included in the model, several factors also significantly influenced the odds of long COVID. Female sex, older age, living in an urban area, infection during Delta or Omicron waves, higher level of COVID-19 severity during the acute COVID-19 phase, reinfection, and positive antibody after infection were associated with higher odds of long COVID. Compared to non-Hispanic Whites, the odds of getting diagnosed with long COVID were higher in Hispanic patients (aOR, 1.07 [95% CI, 1.01–1.13]) and lower in non-Hispanic Blacks (aOR, 0.87 [95% CI, .82–.91]). Some preexisting diseases—including rheumatological disease, obesity, depression, and malnutrition—were also associated with higher odds of long COVID. Diseases associated with lower odds of long COVID included psychosis, diabetes, dementia, heart failure, cancer, hemiplegia or paraplegia, human immunodeficiency virus (HIV) infection, hypertension, and solid organ transplant. Some medications taken before the infection or administered during COVID-19 hospitalization were associated with higher odds, including systemic corticosteroids, remdesivir, immunosuppressants, and therapeutic agents for COVID-19. The anticoagulant and vasopressor were associated with a lower risk of long COVID. History of tobacco use and history of substance abuse were associated with lower odds of developing long COVID after survival from acute COVID-19 (aOR, 0.84 [95% CI, .78–.90] and 0.7 [95% CI, .64–.77], respectively). Full vaccination or having a booster were related to lower odds of long COVID (aOR, 0.94 [95% CI, .90–.98] and 0.94 [95% CI, .90–.98], respectively). Results on demographic, socioeconomic, and geographical covariates are presented in Figure 3. All results of the primary analysis are summarized in Supplementary Table 4.

### Subgroup Analysis

Among patients with CLD, similar to the analysis on the whole cohort, female sex, older age, living in an urban area, infection during Delta or Omicron wave, higher level of severity, reinfection, and positive antibody status were related to higher odds, and tobacco use and substance abuse disorder to lower odds, of long COVID diagnosis. Rheumatologic disease, depression, obesity, and malnutrition were associated with high odds of long COVID. However, vaccination status (completion of primary series or receipt of 1 or more booster doses) did not show a significant effect on long COVID (aOR, 1.04 [95% CI, .96–1.13] and 0.99 [95% CI, .92–1.07], respectively). For patients without CLD before COVID-19, both full vaccination and boosted status were related to a lower risk for long COVID compared to not fully vaccinated (aOR, 0.91 [95% CI, .86–.96] and 0.92 [95% CI, .87–.97], respectively; Supplementary Tables 5 and 6).

### Sensitivity Analysis

First, the follow-up period was <6 months for 10 215 patients in our cohort who were diagnosed with long COVID within the 6-month period. After removing these patients, repeat analyses on the remaining 1 195 806 patients with the same follow-up demonstrated similar risk factors for long COVID (Supplementary Table 7). Second, regarding the concern on underreported long COVID cases, after randomly removing 50% of the confirmed long COVID cases from the cohort, analyses in this underdiagnosis scenario also presented similar results (Supplementary Table 8). Third, sensitivity analysis on the potential competing risk impact of death also showed similar results to the primary analysis (Supplementary Table 9). Overall, our primary analysis results were robust.

## DISCUSSION

In this retrospective cohort study of adult patients diagnosed with COVID-19 who survived to follow-up, we evaluated the association between preexisting CLD and long COVID, and identified the risk factors for developing long COVID among adult patients with CLD.

A prediction model based on N3C data identified CLD as one of the explanatory features for the incidence of long COVID [23]. A cohort study in the Kingdom of Saudi Arabia showed that preexisting lung disease was a predictor for dyspnea and was associated with higher risk for chronic fatigue between 6 weeks and 6 months after hospital discharge in patients with COVID-19 [24]. Our results came to the same conclusion. After adjusting for the confounders, we demonstrated a significant association between preexisting CLD and higher odds of long COVID. We also found that patients were more likely to develop long COVID if they were female, at older ages, or living in an urban area. The effects of sex and age are consistent with previous studies [21, 25]. The association with living in an urban area could be partially explained by the fact that more air pollution in urban area contributes to lung disease [26]. It is also potentially confounded by the fact that people living in urban areas may have more access to healthcare resources and may be more likely to be diagnosed [27]. In addition, some risk factors, such as positive antibody after infection, reinfection, rheumatologic disease, depression, obesity, and malnutrition, may be related to highly activated immune cells and elevated expression of cytokines [28–33]. The higher odds of long COVID could be explained by the immune and inflammatory status under these conditions. Our findings on COVID-19 waves differ from a Spanish retrospective study (N = 325) and a Brazilian case-control study (N = 7051), which identified infection during Delta and Omicron waves as protective factors against long COVID [34, 35]. This discrepancy highlights the importance of conducting the research in different patient populations to investigate the effects of COVID-19 waves.

This study also finds some protective factors against long COVID, such as HIV infection, tobacco use, substance abuse, and vaccination. Existing evidence suggests that immunosuppression could help tamper the cytokine storm or other aspects of the inflammatory response and thus HIV, characterized by immunodeficiency, is shown to have protective effects against long COVID [36]. In terms of tobacco use, studies have shown that smokers are less likely to seek healthcare help for respiratory symptoms and long COVID might be underdiagnosed in this population [37, 38]. Similarly, help-seeking behavior is less frequent in people with substance abuse [39]. This may account for the counterintuitive results that tobacco use and substance abuse were associated with lower odds for long COVID diagnosis. Our results concur with a systematic review and meta-analysis showing that vaccines have a beneficial effect on reducing the risk of long COVID [40]. However, the effects of vaccines were not statistically significant among those with CLD. The adverse impacts of lung disease potentially offset the benefits of vaccination in the CLD subgroup.

Our study is novel in assessing long COVID in a large patient cohort over 3 consecutive COVID-19 waves. Also, we used long COVID diagnosis based on *ICD-10* codes for long COVID (U09.9) to evaluate the outcome, instead of based on persistent PCCs. Use of the *ICD-10* code was a unified way to define the response variable and made it easier to compare the results across studies using the same criteria for the response variable. Another notable advantage of our approach was the comprehensive integration of covariates associated with socioeconomic conditions, geographical influences, and preexisting or concurrent medication use during the COVID-19 pandemic. This not only allowed us to further mitigate potential biases but also facilitated exploration of potential associations with factors such as healthcare accessibility, predominant SARS-CoV-2 variants, vaccination status, and disease severity. These aspects contributed to the robustness and depth of our model.

Our study on long COVID has some limitations. There is potential for underreporting of the long COVID diagnosis and some of the chronic conditions in the dataset. Only 1.2% of our cohort had a long COVID diagnosis on record during the follow-up, a much lower proportion compared to that in other studies. A survey in Italy showed that 28.9% of COVID-19 patients reported persistent symptoms >4 weeks from the onset of infection [41]. The Census Bureau's Household Pulse Survey showed that the prevalence of long COVID decreased to 6% among US adults in June 2023 [42]. Unawareness of long COVID and few show-ups at clinic for persistent symptoms in the early stage of the pandemic account for the underestimated long COVID cases in our study. Nevertheless, sensitivity analysis supports the robustness of the results to the underreporting issue. In addition, the independent and dependent variables are constructed based on the concept sets (*ICD-10* codes, etc) that are dependent on clinician and data coder inputs; data from different sites are potentially heterogeneous. There was no effort as part of this project in verifying these data at the site level as the N3C provides a limited data set with no potential reidentification of patients and no potential for chart review. We suggest improving the dataset by establishing clear definitions for certain variables and exploring interactions between diseases and medication history to improve accuracy. Despite these limitations, our study provides valuable insights into potential risk factors associated with long COVID.

## CONCLUSIONS

Preexisting CLD before COVID-19 is a risk factor for long COVID within 6 months after the acute phase of illness. Patients with CLD should be closely monitored following acute COVID-19, in order to identify long COVID in a timely manner and offer treatment. Prevention of reinfection, nutrition support, and chronic disease management may reduce the risk.

## Supplementary Material

ofae424_Supplementary_Data

## Supplementary Data

Supplementary materials are available at *Open Forum Infectious Diseases* online. Consisting of data provided by the authors to benefit the reader, the posted materials are not copyedited and are the sole responsibility of the authors, so questions or comments should be addressed to the corresponding author.

## Notes

***Acknowledgments*.** This research was possible because of the patients whose information is included within the data and the organizations (https://ncats.nih.gov/n3c/resources/data-contribution/data-transfer-agreement-signatories) and scientists who have contributed to the ongoing development of this community resource (https://doi.org/10.1093/jamia/ocaa196). R. D. is funded by the National Institute of General Medical Sciences, Great Plains Institutional Development Award Network (IDeA) for Clinical and Translational Research (CTR), Great Plains CTR Health Informatics Research Scholar Program (U54 GM115458).

***Author contributions.*** All authors made a significant contribution to the work reported. All authors took part in drafting, revising, or critically reviewing the article, whether in the conception of study design, execution, acquisition of data, analysis, and interpretation of the results. All authors gave final approval of the version to be published and have agreed on the journal to which the article has been submitted and agreed to be accountable for all aspects of the work.

***Patient consent.*** The National Institutes of Health (NIH) National COVID Cohort Collaborative (N3C) Data Utilization Request Approval Committee approved the data utilization request of this project (RP-5677B5), which is approved under the authority of the NIH Institutional Review Board (IRB) and with Johns Hopkins University School of Medicine serving as a central IRB. The study protocol was obtained from the University of Nebraska Medical Center (0175–21-EP, 3/10/2021). The N3C data transfer to the National Center for Advancing Translational Sciences (NCATS) is performed under a Johns Hopkins University Reliance Protocol #IRB00249128 or individual site agreements with NIH. The N3C Data Enclave is managed under the authority of the NIH; information can be found at https://ncats.nih.gov/n3c/resources. No informed consent was obtained because the study used a limited data set.

***Data availability.*** The N3C Enclave is available for public research use. Institutions must have a signed data use agreement executed with the US NCATS to access data. Investigators must complete mandatory training and submit a data use request to N3C. To request N3C data access, follow instructions at https://covid.cd2h.org/onboarding.

***Financial support*.** The analyses described in this publication were conducted with data or tools accessed through the NCATS N3C Data Enclave (https://covid.cd2h.org) and N3C Attribution and Publication Policy v 1.2-2020-08-25b supported by NCATS (U24 TR002306, Axle Informatics Subcontract: NCATS-P00438-B), and the National Institute of General Medical Sciences (U54 GM115458 and U54GM104942), which funds the Great Plains IDeA-CTR Network, GP-CTR Health Informatics Research Scholar Program, and the West Virginia Clinical and Translational Science Institute.

## APPENDIX

We gratefully acknowledge the following core contributors to N3C:

Adam B. Wilcox, Adam M. Lee, Alexis Graves, Alfred (Jerrod) Anzalone, Amin Manna, Amit Saha, Amy Olex, Andrea Zhou, Andrew E. Williams, Andrew Southerland, Andrew T. Girvin, Anita Walden, Anjali A. Sharathkumar, Benjamin Amor, Benjamin Bates, Brian Hendricks, Brijesh Patel, Caleb Alexander, Carolyn Bramante, Cavin Ward-Caviness, Charisse Madlock-Brown, Christine Suver, Christopher Chute, Christopher Dillon, Chunlei Wu, Clare Schmitt, Cliff Takemoto, Dan Housman, Davera Gabriel, David A. Eichmann, Diego Mazzotti, Don Brown, Eilis Boudreau, Elaine Hill, Elizabeth Zampino, Emily Carlson Marti, Emily R. Pfaff, Evan French, Farrukh M. Koraishy, Federico Mariona, Fred Prior, George Sokos, Greg Martin, Harold Lehmann, Heidi Spratt, Hemalkumar Mehta, Hongfang Liu, Hythem Sidky, J. W. Awori Hayanga, Jami Pincavitch, Jaylyn Clark, Jeremy Richard Harper, Jessica Islam, Jin Ge, Joel Gagnier, Joel H. Saltz, Joel Saltz, Johanna Loomba, John Buse, Jomol Mathew, Joni L. Rutter, Julie A. McMurry, Justin Guinney, Justin Starren, Karen Crowley, Katie Rebecca Bradwell, Kellie M. Walters, Ken Wilkins, Kenneth R. Gersing, Kenrick Dwain Cato, Kimberly Murray, Kristin Kostka, Lavance Northington, Lee Allan Pyles, Leonie Misquitta, Lesley Cottrell, Lili Portilla, Mariam Deacy, Mark M. Bissell, Marshall Clark, Mary Emmett, Mary Morrison Saltz, Matvey B. Palchuk, Melissa A. Haendel, Meredith Adams, Meredith Temple-O’Connor, Michael G. Kurilla, Michele Morris, Nabeel Qureshi, Nasia Safdar, Nicole Garbarini, Noha Sharafeldin, Ofer Sadan, Patricia A. Francis, Penny Wung Burgoon, Peter Robinson, Philip R. O. Payne, Rafael Fuentes, Randeep Jawa, Rebecca Erwin-Cohen, Rena Patel, Richard A. Moffitt, Richard L. Zhu, Rishi Kamaleswaran, Robert Hurley, Robert T. Miller, Saiju Pyarajan, Sam G. Michael, Samuel Bozzette, Sandeep Mallipattu, Satyanarayana Vedula, Scott Chapman, Shawn T. O’Neil, Soko Setoguchi, Stephanie S. Hong, Steve Johnson, Tellen D. Bennett, Tiffany Callahan, Umit Topaloglu, Usman Sheikh, Valery Gordon, Vignesh Subbian, Warren A. Kibbe, Wenndy Hernandez, Will Beasley, Will Cooper, William Hillegass, and Xiaohan Tanner Zhang. Details of contributions are available at covid.cd2h.org/core-contributors.

The following institutions’ data are released or pending:

Available: Advocate Health Care Network—UL1TR002389: The Institute for Translational Medicine (ITM) • Aurora Health Care Inc—UL1TR002373: Wisconsin Network For Health Research • Boston University Medical Campus—UL1TR001430: Boston University Clinical and Translational Science Institute • Brown University—U54GM115677: Advance Clinical Translational Research (Advance-CTR) • Carilion Clinic—UL1TR003015: iTHRIV Integrated Translational Health Research Institute of Virginia • Case Western Reserve University—UL1TR002548: The Clinical & Translational Science Collaborative of Cleveland (CTSC) • Charleston Area Medical Center—U54GM104942: West Virginia Clinical and Translational Science Institute (WVCTSI) • Children's Hospital Colorado—UL1TR002535: Colorado Clinical and Translational Sciences Institute • Columbia University Irving Medical Center—UL1TR001873: Irving Institute for Clinical and Translational Research • Dartmouth College—None (Voluntary) • Duke University—UL1TR002553: Duke Clinical and Translational Science Institute • George Washington Children's Research Institute—UL1TR001876: Clinical and Translational Science Institute at Children's National (CTSA-CN) • George Washington University—UL1TR001876: Clinical and Translational Science Institute at Children's National (CTSA-CN) • Harvard Medical School—UL1TR002541: Harvard Catalyst • Indiana University School of Medicine—UL1TR002529: Indiana Clinical and Translational Science Institute • Johns Hopkins University—UL1TR003098: Johns Hopkins Institute for Clinical and Translational Research • Louisiana Public Health Institute—None (Voluntary) • Loyola Medicine—Loyola University Medical Center • Loyola University Medical Center—UL1TR002389: The Institute for Translational Medicine (ITM) • Maine Medical Center—U54GM115516: Northern New England Clinical & Translational Research (NNE-CTR) Network • Mary Hitchcock Memorial Hospital & Dartmouth Hitchcock Clinic—None (Voluntary) • Massachusetts General Brigham—UL1TR002541: Harvard Catalyst • Mayo Clinic Rochester—UL1TR002377: Mayo Clinic Center for Clinical and Translational Science (CCaTS) • Medical University of South Carolina—UL1TR001450: South Carolina Clinical & Translational Research Institute (SCTR) • MITRE Corporation—None (Voluntary) • Montefiore Medical Center—UL1TR002556: Institute for Clinical and Translational Research at Einstein and Montefiore • Nemours—U54GM104941: Delaware CTR ACCEL Program • NorthShore University HealthSystem—UL1TR002389: The Institute for Translational Medicine (ITM) • Northwestern University at Chicago—UL1TR001422: Northwestern University Clinical and Translational Science Institute (NUCATS) • OCHIN—INV-018455: Bill and Melinda Gates Foundation grant to Sage Bionetworks • Oregon Health & Science University—UL1TR002369: Oregon Clinical and Translational Research Institute • Penn State Health Milton S. Hershey Medical Center—UL1TR002014: Penn State Clinical and Translational Science Institute • Rush University Medical Center—UL1TR002389: The Institute for Translational Medicine (ITM) • Rutgers, The State University of New Jersey—UL1TR003017: New Jersey Alliance for Clinical and Translational Science • Stony Brook University—U24TR002306 • The Alliance at the University of Puerto Rico, Medical Sciences Campus—U54GM133807: Hispanic Alliance for Clinical and Translational Research (The Alliance) • The Ohio State University—UL1TR002733: Center for Clinical and Translational Science • The State University of New York at Buffalo—UL1TR001412: Clinical and Translational Science Institute • The University of Chicago—UL1TR002389: The Institute for Translational Medicine (ITM) • The University of Iowa—UL1TR002537: Institute for Clinical and Translational Science • The University of Miami Leonard M. Miller School of Medicine—UL1TR002736: University of Miami Clinical and Translational Science Institute • The University of Michigan at Ann Arbor—UL1TR002240: Michigan Institute for Clinical and Health Research • The University of Texas Health Science Center at Houston—UL1TR003167: Center for Clinical and Translational Sciences (CCTS) • The University of Texas Medical Branch at Galveston—UL1TR001439: The Institute for Translational Sciences • The University of Utah—UL1TR002538: Uhealth Center for Clinical and Translational Science • Tufts Medical Center—UL1TR002544: Tufts Clinical and Translational Science Institute • Tulane University—UL1TR003096: Center for Clinical and Translational Science • The Queens Medical Center—None (Voluntary) • University Medical Center New Orleans—U54GM104940: Louisiana Clinical and Translational Science (LA CaTS) Center • University of Alabama at Birmingham—UL1TR003096: Center for Clinical and Translational Science • University of Arkansas for Medical Sciences—UL1TR003107: UAMS Translational Research Institute • University of Cincinnati—UL1TR001425: Center for Clinical and Translational Science and Training • University of Colorado Denver, Anschutz Medical Campus—UL1TR002535: Colorado Clinical and Translational Sciences Institute • University of Illinois at Chicago—UL1TR002003: UIC Center for Clinical and Translational Science • University of Kansas Medical Center—UL1TR002366: Frontiers: University of Kansas Clinical and Translational Science Institute • University of Kentucky—UL1TR001998: UK Center for Clinical and Translational Science • University of Massachusetts Medical School Worcester—UL1TR001453: The UMass Center for Clinical and Translational Science (UMCCTS) • University Medical Center of Southern Nevada—None (voluntary) • University of Minnesota—UL1TR002494: Clinical and Translational Science Institute • University of Mississippi Medical Center—U54GM115428: Mississippi Center for Clinical and Translational Research (CCTR) • University of Nebraska Medical Center—U54GM115458: Great Plains IDeA-Clinical & Translational Research • University of North Carolina at Chapel Hill—UL1TR002489: North Carolina Translational and Clinical Science Institute • University of Oklahoma Health Sciences Center—U54GM104938: Oklahoma Clinical and Translational Science Institute (OCTSI) • University of Pittsburgh—UL1TR001857: The Clinical and Translational Science Institute (CTSI) • University of Pennsylvania—UL1TR001878: Institute for Translational Medicine and Therapeutics • University of Rochester—UL1TR002001: UR Clinical & Translational Science Institute • University of Southern California—UL1TR001855: The Southern California Clinical and Translational Science Institute (SC CTSI) • University of Vermont—U54GM115516: Northern New England Clinical & Translational Research (NNE-CTR) Network • University of Virginia—UL1TR003015: iTHRIV Integrated Translational health Research Institute of Virginia • University of Washington—UL1TR002319: Institute of Translational Health Sciences • University of Wisconsin-Madison—UL1TR002373: UW Institute for Clinical and Translational Research • Vanderbilt University Medical Center—UL1TR002243: Vanderbilt Institute for Clinical and Translational Research • Virginia Commonwealth University—UL1TR002649: C. Kenneth and Dianne Wright Center for Clinical and Translational Research • Wake Forest University Health Sciences—UL1TR001420: Wake Forest Clinical and Translational Science Institute • Washington University in St. Louis—UL1TR002345: Institute of Clinical and Translational Sciences • Weill Medical College of Cornell University—UL1TR002384: Weill Cornell Medicine Clinical and Translational Science Center • West Virginia University—U54GM104942: West Virginia Clinical and Translational Science Institute (WVCTSI). Submitted: Icahn School of Medicine at Mount Sinai—UL1TR001433: ConduITS Institute for Translational Sciences • The University of Texas Health Science Center at Tyler—UL1TR003167: Center for Clinical and Translational Sciences (CCTS) • University of California, Davis—UL1TR001860: UCDavis Health Clinical and Translational Science Center • University of California, Irvine—UL1TR001414: The UC Irvine Institute for Clinical and Translational Science (ICTS) • University of California, Los Angeles—UL1TR001881: UCLA Clinical Translational Science Institute • University of California, San Diego—UL1TR001442: Altman Clinical and Translational Research Institute • University of California, San Francisco—UL1TR001872: UCSF Clinical and Translational Science Institute • New York University Langone Health Clinical Science Core, Data Resource Core, and PASC Biorepository Core—OTA-21-015A: Post-Acute Sequelae of SARS-CoV-2 Infection Initiative (RECOVER).

Pending: Arkansas Children's Hospital—UL1TR003107: UAMS Translational Research Institute • Baylor College of Medicine—None (Voluntary) • Children's Hospital of Philadelphia—UL1TR001878: Institute for Translational Medicine and Therapeutics • Cincinnati Children's Hospital Medical Center—UL1TR001425: Center for Clinical and Translational Science and Training • Emory University—UL1TR002378: Georgia Clinical and Translational Science Alliance • HonorHealth—None (Voluntary) • Loyola University Chicago—UL1TR002389: The Institute for Translational Medicine (ITM) • Medical College of Wisconsin—UL1TR001436: Clinical and Translational Science Institute of Southeast Wisconsin • MedStar Health Research Institute—None (Voluntary) • Georgetown University—UL1TR001409: The Georgetown-Howard Universities Center for Clinical and Translational Science (GHUCCTS) • MetroHealth—None (Voluntary) • Montana State University—U54GM115371: American Indian/Alaska Native CTR • NYU Langone Medical Center—UL1TR001445: Langone Health's Clinical and Translational Science Institute • Ochsner Medical Center—U54GM104940: Louisiana Clinical and Translational Science (LA CaTS) Center • Regenstrief Institute—UL1TR002529: Indiana Clinical and Translational Science Institute • Sanford Research—None (Voluntary) • Stanford University—UL1TR003142: Spectrum: The Stanford Center for Clinical and Translational Research and Education • The Rockefeller University—UL1TR001866: Center for Clinical and Translational Science • The Scripps Research Institute—UL1TR002550: Scripps Research Translational Institute • University of Florida—UL1TR001427: UF Clinical and Translational Science Institute • University of New Mexico Health Sciences Center—UL1TR001449: University of New Mexico Clinical and Translational Science Center • University of Texas Health Science Center at San Antonio—UL1TR002645: Institute for Integration of Medicine and Science • Yale New Haven Hospital—UL1TR001863: Yale Center for Clinical Investigation.
