# Supplementary material for: Chronic Lung Disease as a Risk Factor for Long COVID in Patients Diagnosed With Coronavirus Disease 2019: A Retrospective Cohort Study
Source: Open Forum Infect Dis. 2024 Aug 23;11(8):ofae424. doi: 10.1093/ofid/ofae424 (PMC11342242; doi:10.1093/ofid/ofae424)
Supplement: ofae424_Supplementary_Data [file ofae424_supplementary_data.docx]

**Supplemental Online Content**

*Xiatong Zhang, A Jerrod Anzalone, Daisy Dai, et al. “Chronic Lung Disease as a Risk Factor for Long COVID in Patients Diagnosed with COVID-19: A Retrospective Cohort Study.”*

Supplementary Table 1. Baseline characteristics of cohort patients

Supplemental Table 2. Baseline characteristics of patients excluded from study cohort

Supplementary Table 3. Long COVID diagnosis by exposure and other covariates

*Incidence of long COVID diagnosis in patient groups by exposure variable and other covariates*

Supplementary Table 4. Risk factors associated with long COVID in the combined cohort

Supplementary Table 5. Risk factors associated with long COVID in patients with CLD

Supplementary Table 6. Risk factors associated with long COVID in patients without CLD

Supplementary Table 7. Risk factors associated with long COVID in patients who completed 6-month follow-up

Supplemental Table 8. Risk factors associated with long COVID in 50% Sampled Cohort

Supplemental Table 9. Risk factors associated with long COVID in Patients with No Post-COVID-19 Death

**Supplemental Table 1. Baseline characteristics of cohort patients**

| **Characteristic** | **Overall,  N = 1,206,021** | **Non-CLD,  N = 1,000,170** | **CLD,  N = 205,851** | **p value** |
| --- | --- | --- | --- | --- |
| Sex |  |  |  | <0.001 |
| Female | 752,215 (62%) | 615,321 (62%) | 136,894 (67%) |  |
| Male | 453,806 (38%) | 384,849 (38%) | 68,957 (33%) |  |
| Age |  |  |  | <0.001 |
| 18-39 | 412,664 (34%) | 363,868 (36%) | 48,796 (24%) |  |
| 40-65 | 558,043 (46%) | 461,751 (46%) | 96,292 (47%) |  |
| >65 | 235,314 (20%) | 174,551 (17%) | 60,763 (30%) |  |
| Race/Ethnicity |  |  |  | <0.001 |
| Non-Hispanic White | 828,070 (69%) | 692,105 (69%) | 135,965 (66%) |  |
| Non-Hispanic Black | 163,464 (14%) | 125,253 (13%) | 38,211 (19%) |  |
| Hispanic | 124,014 (10%) | 104,569 (10%) | 19,445 (9.4%) |  |
| Other | 90,473 (7.5%) | 78,243 (7.8%) | 12,230 (5.9%) |  |
| Urban/Rural Residency |  |  |  | <0.001 |
| Urban | 716,454 (59%) | 598,882 (60%) | 117,572 (57%) |  |
| Urban-Adjacent Rural | 145,895 (12%) | 124,847 (12%) | 21,048 (10%) |  |
| Nonurban-Adjacent Rural | 35,047 (2.9%) | 29,576 (3.0%) | 5,471 (2.7%) |  |
| Missing | 308,625 (26%) | 246,865 (25%) | 61,760 (30%) |  |
| Wave |  |  |  | <0.001 |
| Pre-Delta | 405,958 (34%) | 349,295 (35%) | 56,663 (28%) |  |
| Delta | 348,572 (29%) | 292,271 (29%) | 56,301 (27%) |  |
| Omicron | 451,491 (37%) | 358,604 (36%) | 92,887 (45%) |  |
| Tobacco Usage | 62,084 (5.1%) | 35,607 (3.6%) | 26,477 (13%) | <0.001 |
| Substance Use Disorder | 43,265 (3.6%) | 28,560 (2.9%) | 14,705 (7.1%) | <0.001 |
| COVID-19 Vaccination Status |  |  |  | <0.001 |
| Not Fully Vaccinated | 720,565 (60%) | 597,941 (60%) | 122,624 (60%) |  |
| Fully Vaccinated | 220,615 (18%) | 186,408 (19%) | 34,207 (17%) |  |
| Boosted | 264,841 (22%) | 215,821 (22%) | 49,020 (24%) |  |
| Comorbidities |  |  |  |  |
| Psychosis | 13,433 (1.1%) | 8,566 (0.9%) | 4,867 (2.4%) | <0.001 |
| Diabetes | 202,583 (17%) | 143,005 (14%) | 59,578 (29%) | <0.001 |
| Tuberculosis | 1,414 (0.1%) | 910 (<0.1%) | 504 (0.2%) | <0.001 |
| Liver Disease | 77,946 (6.5%) | 51,283 (5.1%) | 26,663 (13%) | <0.001 |
| Thalassemia | 1,277 (0.1%) | 881 (<0.1%) | 396 (0.2%) | <0.001 |
| Rheumatic Disease | 76,452 (6.3%) | 48,969 (4.9%) | 27,483 (13%) | <0.001 |
| Dementia | 18,153 (1.5%) | 11,887 (1.2%) | 6,266 (3.0%) | <0.001 |
| Congestive Heart Failure | 51,160 (4.2%) | 22,178 (2.2%) | 28,982 (14%) | <0.001 |
| Kidney Disease | 103,827 (8.6%) | 65,301 (6.5%) | 38,526 (19%) | <0.001 |
| Malignant Cancer | 102,547 (8.5%) | 73,124 (7.3%) | 29,423 (14%) | <0.001 |
| Cerebrovascular Disease | 47,140 (3.9%) | 28,398 (2.8%) | 18,742 (9.1%) | <0.001 |
| Peripheral Vascular Disease | 44,882 (3.7%) | 25,621 (2.6%) | 19,261 (9.4%) | <0.001 |
| Hemiplegia/Paraplegia | 11,157 (0.9%) | 6,769 (0.7%) | 4,388 (2.1%) | <0.001 |
| Obesity | 468,807 (39%) | 350,285 (35%) | 118,522 (58%) | <0.001 |
| Coronary Artery Disease | 95,447 (7.9%) | 55,673 (5.6%) | 39,774 (19%) | <0.001 |
| Depression | 221,585 (18%) | 152,355 (15%) | 69,230 (34%) | <0.001 |
| HIV | 7,998 (0.7%) | 5,635 (0.6%) | 2,363 (1.1%) | <0.001 |
| Peptic Ulcer Disease | 17,634 (1.5%) | 10,544 (1.1%) | 7,090 (3.4%) | <0.001 |
| Sickle Cell Disease | 1,979 (0.2%) | 1,051 (0.1%) | 928 (0.5%) | <0.001 |
| Cardiomyopathies | 33,961 (2.8%) | 18,019 (1.8%) | 15,942 (7.7%) | <0.001 |
| Hypertension | 422,347 (35%) | 303,153 (30%) | 119,194 (58%) | <0.001 |
| Solid Organ Transplant | 17,375 (1.4%) | 10,816 (1.1%) | 6,559 (3.2%) | <0.001 |
| Down Syndrome | 178 (<0.1%) | 130 (<0.1%) | 48 (<0.1%) | <0.001 |
| Malnutrition | 247,065 (20%) | 172,306 (17%) | 74,759 (36%) | <0.001 |
| Mineral Deficiency | 24,018 (2.0%) | 15,845 (1.6%) | 8,173 (4.0%) | <0.001 |
| Acute COVID-19 Severity |  |  |  | <0.001 |
| Mild with no ED visit around Covid Index Date | 183,888 (15%) | 148,065 (15%) | 35,823 (17%) |  |
| Mild with ED visit around Covid Index Date | 890,629 (74%) | 759,575 (76%) | 131,054 (64%) |  |
| Moderate with hospitalization around Covid Index Date | 125,848 (10%) | 89,018 (8.9%) | 36,830 (18%) |  |
| Severe with ECMO or IMV during hospitalization around Covid Index Date | 5,656 (0.5%) | 3,512 (0.4%) | 2,144 (1.0%) |  |
| Antibody Positive Post Covid | 19,092 (1.6%) | 15,321 (1.5%) | 3,771 (1.8%) | <0.001 |
| Reinfection | 81,005 (6.7%) | 67,503 (6.7%) | 13,502 (6.6%) | 0.002 |
| Medications |  |  |  |  |
| Systemic Corticosteroids | 424,266 (35%) | 295,249 (30%) | 129,017 (63%) | <0.001 |
| Covid-regimen corticosteroids during Covid hospitalization | 61,919 (5.1%) | 39,637 (4.0%) | 22,282 (11%) | <0.001 |
| Remdesivir during Covid hospitalization | 35,085 (2.9%) | 22,234 (2.2%) | 12,851 (6.2%) | <0.001 |
| Immunosuppressant | 57,739 (4.8%) | 38,558 (3.9%) | 19,181 (9.3%) | <0.001 |
| Antibiotics | 329,899 (27%) | 229,925 (23%) | 99,974 (49%) | <0.001 |
| Anticoagulant | 178,251 (15%) | 111,266 (11%) | 66,985 (33%) | <0.001 |
| Antigout | 27,727 (2.3%) | 19,158 (1.9%) | 8,569 (4.2%) | <0.001 |
| Antiparasite | 141,605 (12%) | 93,818 (9.4%) | 47,787 (23%) | <0.001 |
| Antimetabolite | 17,831 (1.5%) | 11,782 (1.2%) | 6,049 (2.9%) | <0.001 |
| Anti-Covid | 12,085 (1.0%) | 8,793 (0.9%) | 3,292 (1.6%) | <0.001 |
| Estrogen | 33,362 (2.8%) | 24,931 (2.5%) | 8,431 (4.1%) | <0.001 |
| Anti-HIV | 7,197 (0.6%) | 5,263 (0.5%) | 1,934 (0.9%) | <0.001 |
| Fluvoxamine | 1,330 (0.1%) | 984 (<0.1%) | 346 (0.2%) | <0.001 |
| Medroxyprogesterone Acetate | 25,263 (2.1%) | 19,484 (1.9%) | 5,779 (2.8%) | <0.001 |
| Monoclonal Therapies | 21,127 (1.8%) | 15,404 (1.5%) | 5,723 (2.8%) | <0.001 |
| Probenecid | 570 (<0.1%) | 396 (<0.1%) | 174 (<0.1%) | <0.001 |
| Testosterone | 9,723 (0.8%) | 7,633 (0.8%) | 2,090 (1.0%) | <0.001 |
| Vasopressor | 106,669 (8.8%) | 67,573 (6.8%) | 39,096 (19%) | <0.001 |
| Vitamin D | 275,530 (23%) | 195,467 (20%) | 80,063 (39%) | <0.001 |

**Supplemental Table 2. Baseline characteristics of patients excluded from study cohort**

| **Characteristic** | **Overall,  N = 1,687,225** | **Non-CLD,  N = 1,547,490** | **CLD,  N = 139,735** | **p value** |
| --- | --- | --- | --- | --- |
| Sex |  |  |  | <0.001 |
| Female | 896,115 (53%) | 810,963 (52%) | 85,152 (61%) |  |
| Male | 791,110 (47%) | 736,527 (48%) | 54,583 (39%) |  |
| Age |  |  |  | <0.001 |
| 18-39 | 238,537 (14%) | 196,821 (13%) | 41,716 (30%) |  |
| 40-65 | 770,211 (46%) | 729,466 (47%) | 40,745 (29%) |  |
| >65 | 678,477 (40%) | 621,203 (40%) | 57,274 (41%) |  |
| Race/Ethnicity |  |  |  | <0.001 |
| Non-Hispanic White | 177,306 (11%) | 163,935 (11%) | 13,371 (9.6%) |  |
| Non-Hispanic Black | 228,880 (14%) | 200,859 (13%) | 28,021 (20%) |  |
| Hispanic | 1,038,755 (62%) | 951,754 (62%) | 87,001 (62%) |  |
| Other | 242,284 (14%) | 230,942 (15%) | 11,342 (8.1%) |  |
| Urban/Rural Residency |  |  |  | <0.001 |
| Urban | 589,609 (35%) | 528,151 (34%) | 61,458 (44%) |  |
| Urban-Adjacent Rural | 42,764 (2.5%) | 40,276 (2.6%) | 2,488 (1.8%) |  |
| Nonurban-Adjacent Rural | 878,729 (52%) | 813,413 (53%) | 65,316 (47%) |  |
| Missing | 176,123 (10%) | 165,650 (11%) | 10,473 (7.5%) |  |
| Wave |  |  |  | <0.001 |
| Pre-Delta | 400,132 (24%) | 374,572 (24%) | 25,560 (18%) |  |
| Delta | 984,629 (58%) | 892,015 (58%) | 92,614 (66%) |  |
| Omicron | 302,464 (18%) | 280,903 (18%) | 21,561 (15%) |  |
| Tobacco Usage | 51,345 (3.0%) | 35,282 (2.3%) | 16,063 (11%) | <0.001 |
| Substance Use Disorder | 34,729 (2.1%) | 25,911 (1.7%) | 8,818 (6.3%) | <0.001 |
| COVID-19 Vaccination Status |  |  |  | <0.001 |
| Not Fully Vaccinated | 1,269,128 (75%) | 1,166,071 (75%) | 103,057 (74%) |  |
| Fully Vaccinated | 220,398 (13%) | 203,658 (13%) | 16,740 (12%) |  |
| Boosted | 197,699 (12%) | 177,761 (11%) | 19,938 (14%) |  |
| Comorbidities |  |  |  |  |
| Psychosis | 12,004 (0.7%) | 8,752 (0.6%) | 3,252 (2.3%) | <0.001 |
| Diabetes | 1,632,144 (97%) | 1,510,353 (98%) | 121,791 (87%) | <0.001 |
| Tuberculosis | 835 (<0.1%) | 590 (<0.1%) | 245 (0.2%) | <0.001 |
| Liver Disease | 1,642,322 (97%) | 1,514,913 (98%) | 127,409 (91%) | <0.001 |
| Thalassemia | 847 (<0.1%) | 667 (<0.1%) | 180 (0.1%) | <0.001 |
| Rheumatic Disease | 43,125 (2.6%) | 29,881 (1.9%) | 13,244 (9.5%) | <0.001 |
| Dementia | 19,350 (1.1%) | 13,693 (0.9%) | 5,657 (4.0%) | <0.001 |
| Congestive Heart Failure | 33,037 (2.0%) | 15,512 (1.0%) | 17,525 (13%) | <0.001 |
| Kidney Disease | 64,787 (3.8%) | 43,127 (2.8%) | 21,660 (16%) | <0.001 |
| Malignant Cancer | 60,567 (3.6%) | 45,294 (2.9%) | 15,273 (11%) | <0.001 |
| Cerebrovascular Disease | 32,510 (1.9%) | 21,394 (1.4%) | 11,116 (8.0%) | <0.001 |
| Peripheral Vascular Disease | 26,639 (1.6%) | 15,807 (1.0%) | 10,832 (7.8%) | <0.001 |
| Hemiplegia/Paraplegia | 8,898 (0.5%) | 5,867 (0.4%) | 3,031 (2.2%) | <0.001 |
| Obesity | 390,969 (23%) | 322,922 (21%) | 68,047 (49%) | <0.001 |
| Coronary Artery Disease | 62,600 (3.7%) | 39,334 (2.5%) | 23,266 (17%) | <0.001 |
| Depression | 127,313 (7.5%) | 94,603 (6.1%) | 32,710 (23%) | <0.001 |
| HIV | 4,754 (0.3%) | 3,676 (0.2%) | 1,078 (0.8%) | <0.001 |
| Peptic Ulcer Disease | 10,219 (0.6%) | 6,624 (0.4%) | 3,595 (2.6%) | <0.001 |
| Sickle Cell Disease | 1,166 (<0.1%) | 756 (<0.1%) | 410 (0.3%) | <0.001 |
| Cardiomyopathies | 18,960 (1.1%) | 10,692 (0.7%) | 8,268 (5.9%) | <0.001 |
| Hypertension | 296,176 (18%) | 227,504 (15%) | 68,672 (49%) | <0.001 |
| Solid Organ Transplant | 637 (<0.1%) | 94 (<0.1%) | 543 (0.4%) | <0.001 |
| Down Syndrome | 137 (<0.1%) | 111 (<0.1%) | 26 (<0.1%) | <0.001 |
| Malnutrition | 158,946 (9.4%) | 119,862 (7.7%) | 39,084 (28%) | <0.001 |
| Mineral Deficiency | 12,541 (0.7%) | 9,148 (0.6%) | 3,393 (2.4%) | <0.001 |
| Acute COVID-19 Severity |  |  |  | <0.001 |
| Mild with no ED visit around Covid Index Date | 262,160 (16%) | 233,968 (15%) | 28,192 (20%) |  |
| Mild with ED visit around Covid Index Date | 1,285,737 (76%) | 1,204,434 (78%) | 81,303 (58%) |  |
| Moderate with hospitalization around Covid Index Date | 131,567 (7.8%) | 104,000 (6.7%) | 27,567 (20%) |  |
| Severe with ECMO or IMV during hospitalization around Covid Index Date | 7,761 (0.5%) | 5,088 (0.3%) | 2,673 (1.9%) |  |
| Antibody Positive Post Covid | 8,649 (0.5%) | 7,459 (0.5%) | 1,190 (0.9%) | <0.001 |
| Reinfection | 27,317 (1.6%) | 25,044 (1.6%) | 2,273 (1.6%) | 0.8 |
| Medications |  |  |  |  |
| Systemic Corticosteroids | 318,837 (19%) | 245,787 (16%) | 73,050 (52%) | <0.001 |
| Covid-regimen corticosteroids during Covid hospitalization | 65,944 (3.9%) | 48,714 (3.1%) | 17,230 (12%) | <0.001 |
| Remdesivir during Covid hospitalization | 32,134 (1.9%) | 23,085 (1.5%) | 9,049 (6.5%) | <0.001 |
| Immunosuppressant | 1,686,886 (100%) | 1,547,260 (100%) | 139,626 (100%) | <0.001 |
| Antibiotics | 122,597 (7.3%) | 92,501 (6.0%) | 30,096 (22%) | <0.001 |
| Anticoagulant | 22,113 (1.3%) | 13,920 (0.9%) | 8,193 (5.9%) | <0.001 |
| Antigout | 14,601 (0.9%) | 10,612 (0.7%) | 3,989 (2.9%) | <0.001 |
| Antiparasitic | 1,686,962 (100%) | 1,547,250 (100%) | 139,712 (100%) | 0.8 |
| Antimetabolite | 339 (<0.1%) | 286 (<0.1%) | 53 (<0.1%) | <0.001 |
| Anti-Covid | 2,086 (0.1%) | 1,833 (0.1%) | 253 (0.2%) | <0.001 |
| Estrogen | 238 (<0.1%) | 202 (<0.1%) | 36 (<0.1%) | <0.001 |
| Anti-HIV | 1,683,220 (100%) | 1,544,417 (100%) | 138,803 (99%) | <0.001 |
| Fluvoxamine | 845 (<0.1%) | 689 (<0.1%) | 156 (0.1%) | <0.001 |
| Medroxyprogesterone Acetate | 13,442 (0.8%) | 11,245 (0.7%) | 2,197 (1.6%) | <0.001 |
| Monoclonal Therapies | 22,125 (1.3%) | 18,877 (1.2%) | 3,248 (2.3%) | <0.001 |
| Probenecid | 265 (<0.1%) | 201 (<0.1%) | 64 (<0.1%) | <0.001 |
| Testosterone | 5,429 (0.3%) | 4,569 (0.3%) | 860 (0.6%) | <0.001 |
| Vasopressor | 57,873 (3.4%) | 39,943 (2.6%) | 17,930 (13%) | <0.001 |
| Vitamin D | 172,582 (10%) | 134,136 (8.7%) | 38,446 (28%) | <0.001 |

**Supplementary Table 3. Long COVID diagnosis by exposure and other covariates**

| **Factors** | **Patients No. (%)** | **Long Covid No. (%)** |
| --- | --- | --- |
| Long COVID | 14927 (1.2%) | 14927 (100%) |
| Chronic Lung Disease | 205851 (17.1%) | 4314 (2.1%) |
| COPH | 58561 (4.9%) | 1217 (2.1%) |
| Asthma | 135163 (11.2%) | 2797 (2.1%) |
| Bronchiectasis | 6534 (0.5%) | 207 (3.2%) |
| Sex Female | 752215 (62.4%) | 9671 (1.3%) |
| Sex Male | 453806 (37.6%) | 5256 (1.2%) |
| Age group 18-39 | 412664 (34.2%) | 3093 (0.7%) |
| Age group 40-65 | 558043 (46.3%) | 8398 (1.5%) |
| Age group >65 | 235314 (19.5%) | 3436 (1.5%) |
| Race Non-Hispanic White | 828070 (68.7%) | 10384 (1.3%) |
| Race Non-Hispanic Black | 163464 (13.6%) | 1984 (1.2%) |
| Race Hispanic | 124014 (10.3%) | 1563 (1.3%) |
| Race Other | 90473 (7.5%) | 996 (1.1%) |
| Rurality Urban | 716454 (59.4%) | 8429 (1.2%) |
| Rurality Urban-Adjacent Rural | 145895 (12.1%) | 1425 (1%) |
| Rurality Nonurban-Adjacent Rural | 35047 (2.9%) | 357 (1%) |
| Rurality Missing | 308625 (25.6%) | 4716 (1.5%) |
| Wave Pre-Delta | 405958 (33.7%) | 1237 (0.3%) |
| Wave Delta | 348572 (28.9%) | 5995 (1.7%) |
| Wave Omicron | 451491 (37.4%) | 7695 (1.7%) |
| Tobacco Use | 62084 (5.1%) | 938 (1.5%) |
| Substance Abuse | 43265 (3.6%) | 512 (1.2%) |
| Not Fully Vaccinated | 720565 (59.7%) | 9376 (1.3%) |
| Fully Vaccinated | 220615 (18.3%) | 2416 (1.1%) |
| Boosted | 264841 (22%) | 3135 (1.2%) |
| Psychosis | 13433 (1.1%) | 138 (1%) |
| Diabetes | 202583 (16.8%) | 3319 (1.6%) |
| Tuberculosis | 1414 (0.1%) | 21 (1.5%) |
| Liver Disease | 77946 (6.5%) | 1487 (1.9%) |
| Rheumatologic Disease | 76452 (6.3%) | 1650 (2.2%) |
| Dementia | 18153 (1.5%) | 267 (1.5%) |
| Heart Failure | 51160 (4.2%) | 1055 (2.1%) |
| Kidney Disease | 103827 (8.6%) | 1895 (1.8%) |
| Cancer | 102547 (8.5%) | 1531 (1.5%) |
| Cerebrovascular Disease | 47140 (3.9%) | 857 (1.8%) |
| Peripheral Vascular Disease | 44882 (3.7%) | 806 (1.8%) |
| Hemiplegia or Paraplegia | 11157 (0.9%) | 155 (1.4%) |
| Obesity | 468807 (38.9%) | 7892 (1.7%) |
| Coronary Artery Disease | 95447 (7.9%) | 1660 (1.7%) |
| Depression | 221585 (18.4%) | 4070 (1.8%) |
| HIV | 7998 (0.7%) | 94 (1.2%) |
| Peptic Ulcer | 17634 (1.5%) | 356 (2%) |
| Cardiomyopathies | 33961 (2.8%) | 644 (1.9%) |
| HTN | 422347 (35%) | 6775 (1.6%) |
| Transplant | 17375 (1.4%) | 326 (1.9%) |
| Malnutrition | 247065 (20.5%) | 4668 (1.9%) |
| Death | 9516 (0.8%) | 286 (3%) |
| Mild with no ED visit | 183888 (15.2%) | 2194 (1.2%) |
| Mild with ED visit | 890629 (73.8%) | 8504 (1%) |
| Moderate with hospitalization | 125848 (10.4%) | 3486 (2.8%) |
| Severe with ECMO or IMV | 5656 (0.5%) | 743 (13.1%) |
| Antibody Positive Post Covid | 19092 (1.6%) | 498 (2.6%) |
| Reinfection | 81005 (6.7%) | 897 (1.1%) |
| Systemic Corticosteroids | 424266 (35.2%) | 7600 (1.8%) |
| Covid-regimen corticosteroids | 61919 (5.1%) | 2930 (4.7%) |
| Remdesivir during Covid hospitalization | 35085 (2.9%) | 1897 (5.4%) |
| Immunosuppressant | 57739 (4.8%) | 1255 (2.2%) |
| Antibiotics | 329899 (27.4%) | 5405 (1.6%) |
| Anticoagulant | 178251 (14.8%) | 3350 (1.9%) |
| Anti-Covid | 12085 (1%) | 254 (2.1%) |
| Anti-HIV | 7197 (0.6%) | 112 (1.6%) |
| Fluvoxamine | 1330 (0.1%) | 27 (2%) |
| Medroxyprogesterone Acetate | 25263 (2.1%) | 298 (1.2%) |
| Monoclonal Therapies | 21127 (1.8%) | 391 (1.9%) |
| Testosterone | 9723 (0.8%) | 180 (1.9%) |
| Vasopressor | 106669 (8.8%) | 1835 (1.7%) |
| Vitamin D | 275530 (22.8%) | 4749 (1.7%) |

**Supplementary Table 4. Risk factors associated with long COVID in cohort**

| **Variable** | **Odds Ratio** | **95% CI** | **p value** |
| --- | --- | --- | --- |
| Chronic Lung Disease | 1.36 | (1.3, 1.41) | <0.001 |
| Sex |  |  |  |
| Male | Reference |  |  |
| Female | 1.09 | (1.05, 1.13) | <0.001 |
| Age |  |  |  |
| 18-39 | Reference |  |  |
| Age 40-65 | 1.79 | (1.71, 1.87) | <0.001 |
| Age >65 | 1.56 | (1.47, 1.65) | <0.001 |
| Race/Ethnicity |  |  |  |
| Non-Hispanic White | Reference |  |  |
| Non-Hispanic Black | 0.87 | (0.82, 0.91) | <0.001 |
| Hispanic | 1.07 | (1.01, 1.13) | 0.015 |
| Other | 1.01 | (0.94, 1.08) | 0.811 |
| Urban/Rural Residency |  |  |  |
| Urban | Reference |  |  |
| Urban-Adjacent Rural | 0.79 | (0.75, 0.84) | <0.001 |
| Nonurban-Adjacent Rural | 0.78 | (0.7, 0.87) | <0.001 |
| Missing | 1.2 | (1.16, 1.25) | <0.001 |
| Wave |  |  |  |
| Pre-Delta | Reference |  |  |
| Delta | 5.81 | (5.46, 6.18) | <0.001 |
| Omicron | 6.03 | (5.68, 6.42) | <0.001 |
| Tobacco Use | 0.84 | (0.78, 0.9) | <0.001 |
| Substance Abuse | 0.7 | (0.64, 0.77) | <0.001 |
| Vaccination Status |  |  |  |
| Not Fully Vaccinated | Reference |  |  |
| Fully Vaccinated | 0.94 | (0.9, 0.98) | 0.008 |
| Boosted | 0.94 | (0.9, 0.98) | 0.002 |
| Comorbidities |  |  |  |
| Psychosis | 0.62 | (0.52, 0.74) | <0.001 |
| Diabetes | 0.91 | (0.87, 0.96) | <0.001 |
| Tuberculosis | 0.85 | (0.55, 1.32) | 0.467 |
| Liver Disease | 1.05 | (0.99, 1.11) | 0.086 |
| Rheumatologic Disease | 1.17 | (1.11, 1.24) | <0.001 |
| Dementia | 0.86 | (0.76, 0.98) | 0.019 |
| Heart Failure | 0.9 | (0.83, 0.98) | 0.014 |
| Kidney Disease | 0.96 | (0.91, 1.02) | 0.229 |
| Cancer | 0.83 | (0.79, 0.88) | <0.001 |
| Cerebrovascular Disease | 1.05 | (0.98, 1.14) | 0.18 |
| Peripheral Vascular Disease | 0.93 | (0.86, 1.01) | 0.082 |
| Hemiplegia or Paraplegia | 0.62 | (0.52, 0.73) | <0.001 |
| Obesity | 1.32 | (1.28, 1.37) | <0.001 |
| Coronary Artery Disease | 0.97 | (0.91, 1.03) | 0.32 |
| Depression | 1.29 | (1.24, 1.34) | <0.001 |
| HIV Infection | 0.79 | (0.64, 0.99) | 0.037 |
| Peptic Ulcer | 1.02 | (0.92, 1.14) | 0.7 |
| Cardiomyopathies | 1.05 | (0.95, 1.15) | 0.321 |
| Hypertension | 0.94 | (0.9, 0.98) | 0.002 |
| Solid Organ Transplant | 0.68 | (0.59, 0.77) | <0.001 |
| Malnutrition | 1.24 | (1.2, 1.29) | <0.001 |
| Acute COVID-19 Severity |  |  |  |
| Mild with no ED visit | Reference |  |  |
| Mild with ED visit | 1.25 | (1.2, 1.32) | <0.001 |
| Moderate with hospitalization | 2.09 | (1.97, 2.22) | <0.001 |
| Severe with ECMO or IMV | 8.86 | (7.96, 9.86) | <0.001 |
| Antibody Positive Post Covid | 2.02 | (1.83, 2.22) | <0.001 |
| Reinfection post Covid | 1.12 | (1.04, 1.2) | 0.002 |
| Medications |  |  |  |
| Systemic Corticosteroids | 1.2 | (1.16, 1.25) | <0.001 |
| Covid-regimen corticosteroids | 1.57 | (1.45, 1.7) | <0.001 |
| Remdesivir during Covid hospitalization | 1.58 | (1.47, 1.7) | <0.001 |
| Immunosuppressant | 1.1 | (1.03, 1.18) | 0.007 |
| Antibiotics | 1.04 | (1, 1.08) | 0.03 |
| Anticoagulant | 0.78 | (0.74, 0.82) | <0.001 |
| Anti-Covid | 1.32 | (1.15, 1.52) | <0.001 |
| Anti-HIV | 1.19 | (0.97, 1.45) | 0.096 |
| Fluvoxamine | 1.32 | (0.9, 1.94) | 0.157 |
| Medroxyprogesterone Acetate | 0.91 | (0.81, 1.02) | 0.105 |
| Monoclonal Therapies | 1.05 | (0.94, 1.18) | 0.356 |
| Testosterone | 1.16 | (0.99, 1.35) | 0.059 |
| Vasopressor | 0.87 | (0.82, 0.92) | <0.001 |
| Vitamin D | 1.01 | (0.97, 1.05) | 0.786 |

**Supplementary Table 5. Risk factors associated with long COVID in patients with CLD**

| **Variable** | **Odds Ratio** | **95% CI** | **p value** |
| --- | --- | --- | --- |
| Sex |  |  |  |
| Male | Reference |  |  |
| Female | 1.13 | (1.06, 1.22) | <0.001 |
| Age |  |  |  |
| 18-39 | Reference |  |  |
| Age 40-65 | 1.59 | (1.45, 1.74) | <0.001 |
| Age >65 | 1.31 | (1.18, 1.47) | <0.001 |
| Race/Ethnicity |  |  |  |
| Non-Hispanic White | Reference |  |  |
| Non-Hispanic Black | 0.87 | (0.8, 0.95) | 0.002 |
| Hispanic | 0.9 | (0.81, 1.01) | 0.085 |
| Other | 1.02 | (0.89, 1.16) | 0.787 |
| Urban/Rural Residency |  |  |  |
| Urban | Reference |  |  |
| Urban-Adjacent Rural | 0.88 | (0.79, 0.98) | 0.024 |
| Nonurban-Adjacent Rural | 0.68 | (0.55, 0.85) | <0.001 |
| Missing | 1.06 | (0.99, 1.14) | 0.089 |
| Wave |  |  |  |
| Pre-Delta | Reference |  |  |
| Delta | 5.61 | (4.95, 6.35) | <0.001 |
| Omicron | 5.71 | (5.05, 6.44) | <0.001 |
| Tobacco Use | 0.9 | (0.82, 0.99) | 0.032 |
| Substance Abuse | 0.66 | (0.57, 0.76) | <0.001 |
| Vaccination Status |  |  |  |
| Not Fully Vaccinated | Reference |  |  |
| Fully Vaccinated | 1.04 | (0.96, 1.13) | 0.365 |
| Boosted | 0.99 | (0.92, 1.07) | 0.811 |
| Comorbidities |  |  |  |
| Psychosis | 0.72 | (0.57, 0.91) | 0.006 |
| Diabetes | 0.91 | (0.85, 0.98) | 0.015 |
| Tuberculosis | 0.82 | (0.43, 1.55) | 0.537 |
| Liver Disease | 1.03 | (0.94, 1.13) | 0.515 |
| Rheumatologic Disease | 1.15 | (1.06, 1.26) | 0.001 |
| Dementia | 0.83 | (0.69, 1.01) | 0.063 |
| Heart Failure | 0.89 | (0.79, 0.99) | 0.029 |
| Kidney Disease | 1 | (0.91, 1.1) | 0.99 |
| Cancer | 0.89 | (0.81, 0.97) | 0.011 |
| Cerebrovascular Disease | 1.04 | (0.93, 1.17) | 0.467 |
| Peripheral Vascular Disease | 0.99 | (0.88, 1.1) | 0.835 |
| Hemiplegia or Paraplegia | 0.61 | (0.48, 0.78) | <0.001 |
| Obesity | 1.22 | (1.14, 1.3) | <0.001 |
| Coronary Artery Disease | 1.03 | (0.94, 1.12) | 0.58 |
| Depression | 1.17 | (1.1, 1.25) | <0.001 |
| HIV Infection | 0.79 | (0.56, 1.11) | 0.175 |
| Peptic Ulcer | 1.18 | (1.02, 1.37) | 0.028 |
| Cardiomyopathies | 1.08 | (0.95, 1.22) | 0.262 |
| Hypertension | 0.92 | (0.85, 0.99) | 0.032 |
| Solid Organ Transplant | 0.77 | (0.64, 0.92) | 0.005 |
| Malnutrition | 1.22 | (1.14, 1.3) | <0.001 |
| Acute COVID-19 Severity |  |  |  |
| Mild with no ED visit | Reference |  |  |
| Mild with ED visit | 1.01 | (0.92, 1.11) | 0.876 |
| Moderate with hospitalization | 1.53 | (1.36, 1.72) | <0.001 |
| Severe with ECMO or IMV | 6.14 | (5.1, 7.38) | <0.001 |
| Antibody Positive Post Covid | 1.75 | (1.47, 2.1) | <0.001 |
| Reinfection post Covid | 1.23 | (1.09, 1.39) | <0.001 |
| Medications |  |  |  |
| Systemic Corticosteroids | 1.2 | (1.12, 1.3) | <0.001 |
| Covid-regimen corticosteroids | 1.59 | (1.38, 1.82) | <0.001 |
| Remdesivir during Covid hospitalization | 1.47 | (1.31, 1.66) | <0.001 |
| Immunosuppressant | 1.1 | (0.98, 1.23) | 0.092 |
| Antibiotics | 1 | (0.93, 1.06) | 0.904 |
| Anticoagulant | 0.78 | (0.72, 0.85) | <0.001 |
| Anti-Covid | 1.43 | (1.15, 1.78) | 0.002 |
| Anti-HIV | 1.33 | (0.97, 1.84) | 0.077 |
| Fluvoxamine | 1.96 | (1.16, 3.32) | 0.012 |
| Medroxyprogesterone Acetate | 0.83 | (0.68, 1.02) | 0.082 |
| Monoclonal Therapies | 1.08 | (0.9, 1.3) | 0.407 |
| Testosterone | 1.07 | (0.8, 1.42) | 0.644 |
| Vasopressor | 0.88 | (0.81, 0.96) | 0.003 |
| Vitamin D | 1.05 | (0.98, 1.13) | 0.166 |

**Supplementary Table 6. Risk factors associated with long COVID in patients without CLD**

| **Variable** | **Odds Ratio** | **95% CI** | **p value** |
| --- | --- | --- | --- |
| Sex |  |  |  |
| Male | Reference |  |  |
| Female | 1.09 | (1.04, 1.14) | <0.001 |
| Age |  |  |  |
| 18-39 | Reference |  |  |
| Age 40-65 | 1.83 | (1.74, 1.93) | <0.001 |
| Age >65 | 1.62 | (1.52, 1.74) | <0.001 |
| Race/Ethnicity |  |  |  |
| Non-Hispanic White | Reference |  |  |
| Non-Hispanic Black | 0.86 | (0.81, 0.92) | <0.001 |
| Hispanic | 1.12 | (1.05, 1.19) | <0.001 |
| Other | 1 | (0.93, 1.08) | 0.965 |
| Urban/Rural Residency |  |  |  |
| Urban | Reference |  |  |
| Urban-Adjacent Rural | 0.77 | (0.71, 0.82) | <0.001 |
| Nonurban-Adjacent Rural | 0.82 | (0.72, 0.93) | 0.002 |
| Missing | 1.26 | (1.21, 1.32) | <0.001 |
| Wave |  |  |  |
| Pre-Delta | Reference |  |  |
| Delta | 5.84 | (5.43, 6.27) | <0.001 |
| Omicron | 6.12 | (5.7, 6.58) | <0.001 |
| Tobacco Use | 0.79 | (0.71, 0.88) | <0.001 |
| Substance Abuse | 0.74 | (0.65, 0.84) | <0.001 |
| Vaccination Status |  |  |  |
| Not Fully Vaccinated | Reference |  |  |
| Fully Vaccinated | 0.91 | (0.86, 0.96) | <0.001 |
| Boosted | 0.92 | (0.87, 0.97) | 0.001 |
| Comorbidities |  |  |  |
| Psychosis | 0.55 | (0.43, 0.71) | <0.001 |
| Diabetes | 0.92 | (0.87, 0.97) | 0.003 |
| Tuberculosis | 0.91 | (0.5, 1.65) | 0.752 |
| Liver Disease | 1.08 | (1, 1.17) | 0.043 |
| Rheumatologic Disease | 1.2 | (1.11, 1.29) | <0.001 |
| Dementia | 0.89 | (0.75, 1.05) | 0.158 |
| Heart Failure | 1.05 | (0.93, 1.19) | 0.461 |
| Kidney Disease | 0.96 | (0.88, 1.03) | 0.265 |
| Cancer | 0.8 | (0.74, 0.86) | <0.001 |
| Cerebrovascular Disease | 1.07 | (0.96, 1.18) | 0.244 |
| Peripheral Vascular Disease | 0.89 | (0.79, 1) | 0.049 |
| Hemiplegia or Paraplegia | 0.65 | (0.51, 0.81) | <0.001 |
| Obesity | 1.34 | (1.28, 1.4) | <0.001 |
| Coronary Artery Disease | 0.94 | (0.86, 1.02) | 0.128 |
| Depression | 1.36 | (1.29, 1.43) | <0.001 |
| HIV Infection | 0.79 | (0.59, 1.05) | 0.104 |
| Peptic Ulcer | 0.88 | (0.75, 1.04) | 0.134 |
| Cardiomyopathies | 1 | (0.87, 1.15) | 0.983 |
| Hypertension | 0.94 | (0.9, 0.99) | 0.013 |
| Solid Organ Transplant | 0.61 | (0.5, 0.74) | <0.001 |
| Malnutrition | 1.26 | (1.2, 1.32) | <0.001 |
| Acute COVID-19 Severity |  |  |  |
| Mild with no ED visit | Reference |  |  |
| Mild with ED visit | 1.35 | (1.28, 1.43) | <0.001 |
| Moderate with hospitalization | 2.32 | (2.15, 2.49) | <0.001 |
| Severe with ECMO or IMV | 10.1 | (8.85, 11.52) | <0.001 |
| Antibody Positive Post Covid | 2.14 | (1.91, 2.4) | <0.001 |
| Reinfection post Covid | 1.08 | (0.99, 1.18) | 0.07 |
| Medications |  |  |  |
| Systemic Corticosteroids | 1.18 | (1.13, 1.23) | <0.001 |
| Covid-regimen corticosteroids | 1.62 | (1.47, 1.79) | <0.001 |
| Remdesivir during Covid hospitalization | 1.66 | (1.51, 1.82) | <0.001 |
| Immunosuppressant | 1.1 | (1.01, 1.21) | 0.033 |
| Antibiotics | 1.06 | (1.02, 1.11) | 0.007 |
| Anticoagulant | 0.8 | (0.75, 0.85) | <0.001 |
| Anti-Covid | 1.24 | (1.04, 1.48) | 0.018 |
| Anti-HIV | 1.1 | (0.85, 1.43) | 0.469 |
| Fluvoxamine | 0.91 | (0.51, 1.63) | 0.76 |
| Medroxyprogesterone Acetate | 0.93 | (0.81, 1.08) | 0.353 |
| Monoclonal Therapies | 1.02 | (0.89, 1.18) | 0.741 |
| Testosterone | 1.2 | (1, 1.43) | 0.05 |
| Vasopressor | 0.87 | (0.81, 0.93) | <0.001 |
| Vitamin D | 0.98 | (0.93, 1.03) | 0.407 |

**Supplementary Table 7. Risk factors associated with long COVID in patients who completed 6-month follow-up**

| **Variable** | **Odds Ratio** | **95% CI** | **p value** |
| --- | --- | --- | --- |
| Chronic Lung Disease | 1.36 | (1.29, 1.42) | <0.001 |
| Sex |  |  |  |
| Male | Reference |  |  |
| Female | 1.14 | (1.09, 1.19) | <0.001 |
| Age |  |  |  |
| 18-39 | Reference |  |  |
| Age 40-65 | 1.87 | (1.78, 1.97) | <0.001 |
| Age >65 | 1.51 | (1.41, 1.61) | <0.001 |
| Race/Ethnicity |  |  |  |
| Non-Hispanic White | Reference |  |  |
| Non-Hispanic Black | 0.88 | (0.83, 0.93) | <0.001 |
| Hispanic | 1.1 | (1.03, 1.17) | 0.005 |
| Other | 0.98 | (0.91, 1.06) | 0.645 |
| Urban/Rural Residency |  |  |  |
| Urban | Reference |  |  |
| Urban-Adjacent Rural | 0.79 | (0.74, 0.84) | <0.001 |
| Nonurban-Adjacent Rural | 0.77 | (0.68, 0.86) | <0.001 |
| Missing | 0.95 | (0.91, 0.99) | 0.022 |
| Wave |  |  |  |
| Pre-Delta | Reference |  |  |
| Delta | 5.24 | (4.91, 5.59) | <0.001 |
| Omicron | 4.07 | (3.81, 4.34) | <0.001 |
| Tobacco Use | 0.86 | (0.8, 0.93) | <0.001 |
| Substance Abuse | 0.74 | (0.67, 0.82) | <0.001 |
| Vaccination Status |  |  |  |
| Not Fully Vaccinated | Reference |  |  |
| Fully Vaccinated | 1.01 | (0.96, 1.07) | 0.615 |
| Boosted | 1.02 | (0.97, 1.07) | 0.455 |
| Comorbidities |  |  |  |
| Psychosis | 0.62 | (0.51, 0.75) | <0.001 |
| Diabetes | 0.93 | (0.89, 0.98) | 0.007 |
| Tuberculosis | 0.87 | (0.54, 1.42) | 0.589 |
| Liver Disease | 1.05 | (0.99, 1.13) | 0.107 |
| Rheumatologic Disease | 1.19 | (1.12, 1.27) | <0.001 |
| Dementia | 0.84 | (0.73, 0.98) | 0.022 |
| Heart Failure | 0.83 | (0.76, 0.91) | <0.001 |
| Kidney Disease | 0.96 | (0.9, 1.03) | 0.279 |
| Cancer | 0.86 | (0.8, 0.91) | <0.001 |
| Cerebrovascular Disease | 1.06 | (0.98, 1.16) | 0.16 |
| Peripheral Vascular Disease | 0.92 | (0.84, 1.01) | 0.076 |
| Hemiplegia or Paraplegia | 0.58 | (0.48, 0.71) | <0.001 |
| Obesity | 1.4 | (1.35, 1.46) | <0.001 |
| Coronary Artery Disease | 0.97 | (0.91, 1.04) | 0.443 |
| Depression | 1.36 | (1.3, 1.42) | <0.001 |
| HIV Infection | 0.84 | (0.66, 1.08) | 0.17 |
| Peptic Ulcer | 1.06 | (0.94, 1.2) | 0.326 |
| Cardiomyopathies | 1.1 | (0.99, 1.23) | 0.064 |
| Hypertension | 0.97 | (0.93, 1.02) | 0.265 |
| Solid Organ Transplant | 0.65 | (0.56, 0.76) | <0.001 |
| Malnutrition | 1.26 | (1.21, 1.32) | <0.001 |
| Acute COVID-19 Severity |  |  |  |
| Mild with no ED visit | Reference |  |  |
| Mild with ED visit | 1.28 | (1.21, 1.35) | <0.001 |
| Moderate with hospitalization | 2.16 | (2.02, 2.32) | <0.001 |
| Severe with ECMO or IMV | 8.63 | (7.65, 9.73) | <0.001 |
| Antibody Positive Post Covid | 2.07 | (1.87, 2.3) | <0.001 |
| Reinfection post Covid | 1.22 | (1.13, 1.32) | <0.001 |
| Medications |  |  |  |
| Systemic Corticosteroids | 1.26 | (1.21, 1.32) | <0.001 |
| Covid-regimen corticosteroids | 1.46 | (1.34, 1.6) | <0.001 |
| Remdesivir during Covid hospitalization | 1.55 | (1.42, 1.68) | <0.001 |
| Immunosuppressant | 1.13 | (1.04, 1.22) | 0.002 |
| Antibiotics | 1.11 | (1.07, 1.16) | <0.001 |
| Anticoagulant | 0.76 | (0.72, 0.81) | <0.001 |
| Anti-Covid | 0.9 | (0.75, 1.09) | 0.283 |
| Anti-HIV | 0.95 | (0.73, 1.23) | 0.681 |
| Fluvoxamine | 1.39 | (0.91, 2.13) | 0.133 |
| Medroxyprogesterone Acetate | 0.99 | (0.88, 1.13) | 0.92 |
| Monoclonal Therapies | 1.19 | (1.05, 1.34) | 0.006 |
| Testosterone | 1.26 | (1.06, 1.48) | 0.008 |
| Vasopressor | 0.9 | (0.84, 0.95) | <0.001 |
| Vitamin D | 1.11 | (1.07, 1.17) | <0.001 |

**Supplemental Table 8. Risk factors associated with long COVID in 50% Sampled Cohort**

| **Variable** | **Odds Ratio** | **95% CI** | **p value** |
| --- | --- | --- | --- |
| Chronic Lung Disease | 1.37 | (1.3, 1.45) | <0.001 |
| Sex |  |  |  |
| Male | Reference |  |  |
| Female | 1.1 | (1.05, 1.16) | <0.001 |
| Age |  |  |  |
| 18-39 | Reference |  |  |
| Age 40-65 | 1.79 | (1.68, 1.91) | <0.001 |
| Age >65 | 1.55 | (1.43, 1.68) | <0.001 |
| Race/Ethnicity |  |  |  |
| Non-Hispanic White | Reference |  |  |
| Non-Hispanic Black | 0.89 | (0.83, 0.96) | 0.001 |
| Hispanic | 1.09 | (1.01, 1.18) | 0.033 |
| Other | 1.07 | (0.98, 1.18) | 0.129 |
| Urban/Rural Residency |  |  |  |
| Urban | Reference |  |  |
| Urban-Adjacent Rural | 0.81 | (0.75, 0.88) | <0.001 |
| Nonurban-Adjacent Rural | 0.76 | (0.65, 0.89) | <0.001 |
| Missing | 1.18 | (1.12, 1.24) | <0.001 |
| Wave |  |  |  |
| Pre-Delta | Reference |  |  |
| Delta | 5.42 | (4.97, 5.9) | <0.001 |
| Omicron | 5.74 | (5.27, 6.25) | <0.001 |
| Tobacco Use | 0.82 | (0.74, 0.9) | <0.001 |
| Substance Abuse | 0.74 | (0.65, 0.85) | <0.001 |
| Vaccination Status |  |  |  |
| Not Fully Vaccinated | Reference |  |  |
| Fully Vaccinated | 0.94 | (0.88, 1) | 0.047 |
| Boosted | 0.95 | (0.89, 1.01) | 0.088 |
| Comorbidities |  |  |  |
| Psychosis | 0.63 | (0.5, 0.8) | <0.001 |
| Diabetes | 0.9 | (0.84, 0.96) | <0.001 |
| Tuberculosis | 0.97 | (0.54, 1.71) | 0.904 |
| Liver Disease | 1.05 | (0.97, 1.14) | 0.216 |
| Rheumatologic Disease | 1.1 | (1.02, 1.2) | 0.017 |
| Dementia | 0.82 | (0.68, 0.98) | 0.029 |
| Heart Failure | 0.9 | (0.81, 1.01) | 0.083 |
| Kidney Disease | 0.94 | (0.86, 1.02) | 0.115 |
| Cancer | 0.84 | (0.78, 0.91) | <0.001 |
| Cerebrovascular Disease | 1.07 | (0.96, 1.19) | 0.251 |
| Peripheral Vascular Disease | 0.93 | (0.83, 1.04) | 0.186 |
| Hemiplegia or Paraplegia | 0.63 | (0.5, 0.79) | <0.001 |
| Obesity | 1.32 | (1.26, 1.39) | <0.001 |
| Coronary Artery Disease | 1 | (0.91, 1.09) | 0.927 |
| Depression | 1.25 | (1.18, 1.32) | <0.001 |
| HIV Infection | 0.86 | (0.64, 1.16) | 0.324 |
| Peptic Ulcer | 1.08 | (0.93, 1.26) | 0.309 |
| Cardiomyopathies | 1.11 | (0.97, 1.26) | 0.118 |
| Hypertension | 0.93 | (0.87, 0.98) | 0.008 |
| Solid Organ Transplant | 0.69 | (0.58, 0.83) | <0.001 |
| Malnutrition | 1.27 | (1.2, 1.34) | <0.001 |
| Acute COVID-19 Severity |  |  |  |
| Mild with no ED visit | Reference |  |  |
| Mild with ED visit | 1.21 | (1.13, 1.3) | <0.001 |
| Moderate with hospitalization | 2.18 | (2, 2.37) | <0.001 |
| Severe with ECMO or IMV | 8.07 | (6.98, 9.34) | <0.001 |
| Antibody Positive Post Covid | 1.79 | (1.56, 2.06) | <0.001 |
| Reinfection post Covid | 1.13 | (1.03, 1.25) | 0.013 |
| Medications |  |  |  |
| Systemic Corticosteroids | 1.24 | (1.18, 1.31) | <0.001 |
| Covid-regimen corticosteroids | 1.57 | (1.41, 1.76) | <0.001 |
| Remdesivir during Covid hospitalization | 1.5 | (1.36, 1.66) | <0.001 |
| Immunosuppressant | 1.12 | (1.01, 1.23) | 0.029 |
| Antibiotics | 1.04 | (0.98, 1.09) | 0.17 |
| Anticoagulant | 0.79 | (0.73, 0.84) | <0.001 |
| Anti-Covid | 1.11 | (0.91, 1.37) | 0.301 |
| Anti-HIV | 1.18 | (0.89, 1.56) | 0.247 |
| Fluvoxamine | 1.39 | (0.82, 2.36) | 0.227 |
| Medroxyprogesterone Acetate | 0.88 | (0.74, 1.04) | 0.133 |
| Monoclonal Therapies | 1.1 | (0.94, 1.29) | 0.235 |
| Testosterone | 1.17 | (0.94, 1.44) | 0.159 |
| Vasopressor | 0.85 | (0.79, 0.92) | <0.001 |
| Vitamin D | 0.98 | (0.92, 1.03) | 0.42 |

**Supplemental Table 9. Risk factors associated with long COVID in Patients with No Post-COVID-19 Death**

| **Variable** | **Odds Ratio** | **95% CI** | **p value** |
| --- | --- | --- | --- |
| Chronic Lung Disease | 1.36 | (1.31, 1.42) | <0.001 |
| Sex |  |  |  |
| Male | Reference |  |  |
| Female | 1.1 | (1.06, 1.14) | <0.001 |
| Age |  |  |  |
| 18-39 | Reference |  |  |
| Age 40-65 | 1.79 | (1.71, 1.87) | <0.001 |
| Age >65 | 1.54 | (1.45, 1.63) | <0.001 |
| Race/Ethnicity |  |  |  |
| Non-Hispanic White | Reference |  |  |
| Non-Hispanic Black | 0.86 | (0.82, 0.91) | <0.001 |
| Hispanic | 1.08 | (1.02, 1.14) | 0.011 |
| Other | 1.01 | (0.94, 1.08) | 0.759 |
| Urban/Rural Residency |  |  |  |
| Urban | Reference |  |  |
| Urban-Adjacent Rural | 0.79 | (0.74, 0.83) | <0.001 |
| Nonurban-Adjacent Rural | 0.78 | (0.7, 0.87) | <0.001 |
| Missing | 1.21 | (1.16, 1.25) | <0.001 |
| Wave |  |  |  |
| Pre-Delta | Reference |  |  |
| Delta | 5.74 | (5.39, 6.11) | <0.001 |
| Omicron | 5.94 | (5.59, 6.32) | <0.001 |
| Tobacco Use | 0.84 | (0.78, 0.91) | <0.001 |
| Substance Abuse | 0.69 | (0.63, 0.76) | <0.001 |
| Vaccination Status |  |  |  |
| Not Fully Vaccinated | Reference |  |  |
| Fully Vaccinated | 0.94 | (0.9, 0.99) | 0.011 |
| Boosted | 0.94 | (0.9, 0.98) | 0.003 |
| Comorbidities |  |  |  |
| Psychosis | 0.61 | (0.51, 0.73) | <0.001 |
| Diabetes | 0.91 | (0.87, 0.96) | <0.001 |
| Tuberculosis | 0.75 | (0.47, 1.21) | 0.241 |
| Liver Disease | 1.05 | (0.99, 1.11) | 0.123 |
| Rheumatologic Disease | 1.17 | (1.11, 1.24) | <0.001 |
| Dementia | 0.86 | (0.75, 0.98) | 0.025 |
| Heart Failure | 0.9 | (0.82, 0.98) | 0.012 |
| Kidney Disease | 0.95 | (0.9, 1.01) | 0.117 |
| Cancer | 0.83 | (0.78, 0.87) | <0.001 |
| Cerebrovascular Disease | 1.03 | (0.95, 1.12) | 0.422 |
| Peripheral Vascular Disease | 0.92 | (0.85, 1) | 0.06 |
| Hemiplegia or Paraplegia | 0.61 | (0.51, 0.73) | <0.001 |
| Obesity | 1.32 | (1.27, 1.37) | <0.001 |
| Coronary Artery Disease | 0.96 | (0.9, 1.03) | 0.233 |
| Depression | 1.3 | (1.25, 1.35) | <0.001 |
| HIV Infection | 0.79 | (0.63, 0.99) | 0.038 |
| Peptic Ulcer | 1.01 | (0.9, 1.13) | 0.824 |
| Cardiomyopathies | 1.04 | (0.95, 1.15) | 0.388 |
| Hypertension | 0.94 | (0.9, 0.98) | 0.004 |
| Solid Organ Transplant | 0.66 | (0.58, 0.76) | <0.001 |
| Malnutrition | 1.24 | (1.19, 1.29) | <0.001 |
| Acute COVID-19 Severity |  |  |  |
| Mild with no ED visit | Reference |  |  |
| Mild with ED visit | 1.25 | (1.19, 1.32) | <0.001 |
| Moderate with hospitalization | 2.11 | (1.98, 2.24) | <0.001 |
| Severe with ECMO or IMV | 8.88 | (7.96, 9.9) | <0.001 |
| Antibody Positive Post Covid | 2.01 | (1.83, 2.22) | <0.001 |
| Reinfection post Covid | 1.1 | (1.03, 1.18) | 0.008 |
| Medications |  |  |  |
| Systemic Corticosteroids | 1.2 | (1.15, 1.25) | <0.001 |
| Covid-regimen corticosteroids | 1.55 | (1.43, 1.68) | <0.001 |
| Remdesivir during Covid hospitalization | 1.58 | (1.46, 1.7) | <0.001 |
| Immunosuppressant | 1.08 | (1.01, 1.16) | 0.027 |
| Antibiotics | 1.04 | (1, 1.08) | 0.03 |
| Anticoagulant | 0.77 | (0.74, 0.81) | <0.001 |
| Anti-Covid | 1.32 | (1.15, 1.52) | <0.001 |
| Anti-HIV | 1.16 | (0.95, 1.42) | 0.154 |
| Fluvoxamine | 1.34 | (0.91, 1.98) | 0.135 |
| Medroxyprogesterone Acetate | 0.9 | (0.8, 1.02) | 0.091 |
| Monoclonal Therapies | 1.06 | (0.94, 1.18) | 0.336 |
| Testosterone | 1.15 | (0.98, 1.34) | 0.077 |
| Vasopressor | 0.88 | (0.83, 0.93) | <0.001 |
| Vitamin D | 1.01 | (0.97, 1.05) | 0.64 |
